# An agent-based model of cardiac allograft vasculopathy: toward a better understanding of chronic rejection dynamics

**DOI:** 10.3389/fbioe.2023.1190409

**Published:** 2023-09-12

**Authors:** Elisa Serafini, Anna Corti, Diego Gallo, Claudio Chiastra, Xian C. Li, Stefano Casarin

**Affiliations:** ^1^ Polito^BIO^Med Lab, Department of Mechanical and Aerospace Engineering, Politecnico di Torino, Turin, Italy; ^2^ LaSIE, UMR 7356 CNRS, La Rochelle Université, La Rochelle, France; ^3^ Center for Precision Surgery, Houston Methodist Research Institute, Houston, TX, United States; ^4^ Laboratory of Biological Structure Mechanics (LaBS), Department of Chemistry, Materials and Chemical Engineering “Giulio Natta”, Politecnico di Milano, Milan, Italy; ^5^ Immunobiology and Transplant Science Center, Houston Methodist Hospital, Houston, TX, United States; ^6^ Department of Surgery, Weill Cornell Medical College of Cornell University, New York, NY, United States; ^7^ Department of Surgery, Houston Methodist Hospital, Houston, TX, United States

**Keywords:** heart transplant, chronic rejection, cardiac allograft vasculopathy, arterial wall remodeling, coronary artery, computational model, agent-based modeling, sensitivity analysis

## Abstract

Cardiac allograft vasculopathy (CAV) is a coronary artery disease affecting 50% of heart transplant (HTx) recipients, and it is the major cause of graft loss. CAV is driven by the interplay of immunological and non-immunological factors, setting off a cascade of events promoting endothelial damage and vascular dysfunction. The etiology and evolution of tissue pathology are largely unknown, making disease management challenging. So far, *in vivo* models, mostly mouse-based, have been widely used to study CAV, but they are resource-consuming, pose many ethical issues, and allow limited investigation of time points and important biomechanical measurements. Recently, agent-based models (ABMs) proved to be valid computational tools for deciphering mechanobiological mechanisms driving vascular adaptation processes at the cell/tissue level, augmenting cost-effective *in vivo* lab-based experiments, at the same time guaranteeing richness in observation time points and low consumption of resources. We hypothesize that integrating ABMs with lab-based experiments can aid *in vivo* research by overcoming those limitations. Accordingly, this work proposes a bidimensional ABM of CAV in a mouse coronary artery cross-section, simulating the arterial wall response to two distinct stimuli: inflammation and hemodynamic disturbances, the latter considered in terms of low wall shear stress (WSS). These stimuli trigger i) inflammatory cell activation and ii) exacerbated vascular cell activities. Moreover, an extensive analysis was performed to investigate the ABM sensitivity to the driving parameters and inputs and gain insights into the ABM working mechanisms. The ABM was able to effectively replicate a 4-week CAV initiation and progression, characterized by lumen area decrease due to progressive intimal thickening in regions exposed to high inflammation and low WSS. Moreover, the parameter and input sensitivity analysis highlighted that the inflammatory-related events rather than the WSS predominantly drive CAV, corroborating the inflammatory nature of the vasculopathy. The proof-of-concept model proposed herein demonstrated its potential in deepening the pathology knowledge and supporting the *in vivo* analysis of CAV.

## 1 Introduction

Heart transplant (HTx) is the standard of care for patients with refractory end-stage heart disease. More than 5,000 transplants are performed yearly worldwide (Khush et al., 2019; Chambers et al., 2021; Spartalis et al., 2022). Thanks to advances in immunosuppressive therapies and donor–recipient matching criteria, the overall recipient survival has significantly increased in the last two decades (Dharmavaram et al., 2021). While progresses in immunosuppressive drugs markedly improved the HTx short-term outcome, long-term survival is still compromised by the high incidence of cardiac allograft vasculopathy (CAV), which affects 50% of recipients and is the major cause of late mortality (Stehlik et al., 2018; Spartalis et al., 2022). CAV is an inflammatory fibroproliferative disease that affects graft vasculature, manifests as an accelerated form of atherosclerosis, and ultimately leads to organ dysfunction (Chih et al., 2016; Pighi et al., 2020). The pathology is driven by a complex interplay between immunological and non-immunological factors (e.g., hyperlipidemia, ischemia–reperfusion time, biomechanics, and comorbidity). An early inflammatory response promotes endothelial injury and, subsequently, a cascade of maladaptive remodeling processes, involving, among others, exacerbated vascular and inflammatory cellular activities (Chih et al., 2016; Pighi et al., 2020). However, a thorough understanding of the pathology etiology and evolution is lacking.

Current disease management, from diagnosis to treatment, is still controversial and the only curative intervention is re-HTx, recommended in advanced CAV patients with graft impairment (Chih et al., 2016; Pighi et al., 2020; Spartalis et al., 2022). In this context, understanding the multiscale immunological and mechanobiological mechanisms leading to degenerative cellular and molecular events is pivotal to improve prevention, diagnosis, and treatment of CAV.

So far, *in vivo* murine models have been of great importance in gaining valuable insights into CAV pathophysiology (Pober et al., 2021). However, *in vivo* studies are logistically demanding, time-consuming, limited in their ability to efficiently address the multi-scale nature of the disease, pose animal welfare issues, and give access only to a limited number of time points during experimental follow-up, which are instead pivotal to deepen the understanding of the pathology dynamics. In this context, *in silico* approaches have the potential to address some of the *in vivo* study limitations, thus representing a valid support to the experimental research. Computational models have been successfully applied to a broad spectrum of complex pathologies (Barh et al., 2014), including cardiac and vascular diseases (Corti et al., 2021). However, to the best of the authors’ knowledge, CAV has been under-investigated through *in silico* approaches, with previous studies mainly applying computational fluid dynamics (CFD) to elucidate the role of blood flow dynamics (Timmins et al., 2013; Timmins et al., 2015).

Recently, continuum models have been adopted to simulate vascular maladaptive phenomena by accounting for immune cell dynamics in a continuum description (Gierig et al., 2023). In addition, agent-based models (ABMs) have emerged as a promising computational approach to capture cell/tissue mechanobiological processes underlying vascular adaptation (Corti et al., 2021). Specifically, ABMs of vascular remodeling, describing cellular dynamics and cell–cell and cell–environment interactions, were proposed, demonstrating their potentialities in the context of atherosclerosis and restenosis after endovascular/surgical procedures (Corti et al., 2021). Consequently, we hypothesize that studying CAV through the development of an *ad hoc* ABM will provide us with a deeper understanding of the pathology initiation and development, ultimately aiding in improving treatment and prevention.

Accordingly, the current work presents the development of an ABM of CAV initiation and progression along 4 weeks of follow-up in a mouse left coronary artery (LCA) cross-section to provide further understanding in the immunologic and non-immunologic events involved in the pathology initiation and development. We leveraged the versatility of a previously developed ABM, which has demonstrated successful applications in various maladaptive phenomena (Garbey et al., 2017; Corti et al., 2019; Corti et al., 2020; Corti et al., 2022a; Corti et al., 2022b; Corti et al., 2023a; Corti et al., 2023b). By adapting this ABM to investigate the specific pathology of CAV, we efficiently explored unique dynamics and mechanisms of this disease while capitalizing on the proven effectiveness of the computational framework. In addition to the immunological factors, we placed particular emphasis on investigating the impact of different wall shear stress (WSS) patterns on the vascular dynamics. The extensive model study includes a parameter sensitivity analysis and a subsequent input scenario testing to understand the relative weight of the different pathological risk factors and to gain mechanistic insights into CAV initiation and progression.

## 2 Methods

### 2.1 Agent-based model of CAV

A bidimensional (2D) ABM was implemented in MATLAB (R2021b, MathWorks, Natick, MA, USA) to simulate the CAV development and progression in an idealized bilayered mouse LCA cross-section along 4 weeks of follow-up.

In the current work, three cellular/molecular events were assumed to be the major events during the post-HTx period at the cell–tissue level driving CAV formation (Vergani et al., 2013; Kloc and Ghobrial, 2014; Liu et al., 2016). In detail, the events are as follows: i) macrophage (MP) infiltration, mitosis/apoptosis, and chemoattractant diffusion; ii) smooth muscle cell (SMC) mitosis/apoptosis and migration; and iii) extracellular matrix (ECM) production/degradation. Each event was driven by probabilistic rules defined such that, under baseline conditions (i.e., without stimuli), they guarantee homeostasis, while under inflammatory and WSS-based stimuli, they are perturbed, determining the initiation and development of CAV. Due to the stochastic nature of the model, three independent simulations were performed for each investigated scenario (Corti et al., 2022a; Corti et al., 2022b).

**FIGURE 1:**
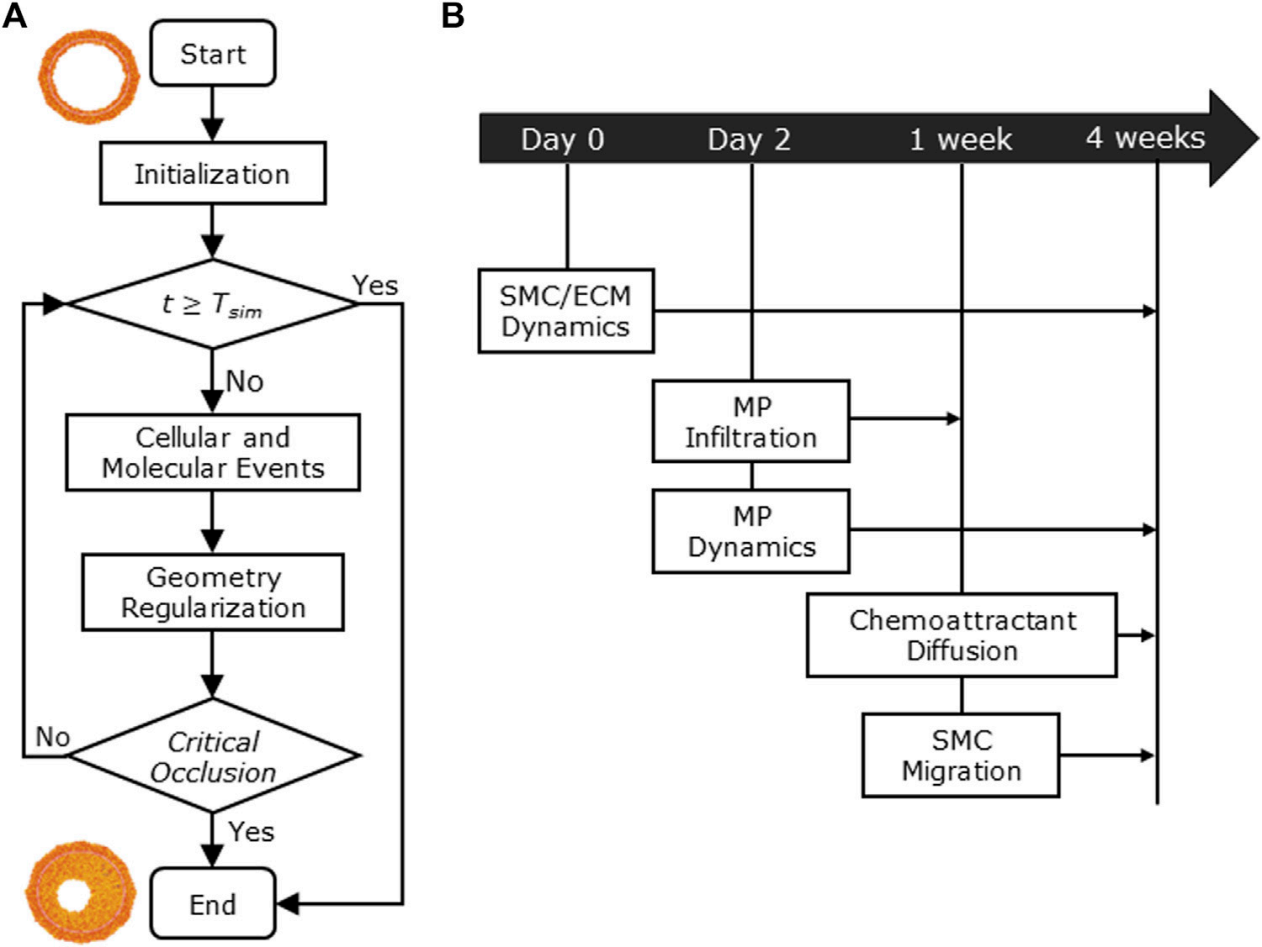
**(A)** Agent-based model (ABM) working mechanism flow chart. **(B)** 4-week timeline of the implemented cellular and molecular events: smooth muscle cell/extracellular matrix (SMC/ECM) dynamics from Day 0 (i.e., heart transplant time) to 4 weeks (i.e., $T_{sim}$ ), macrophage (MP) infiltration from Day 2 to 1 week, MP dynamics from Day 2 to 4 weeks, chemoattractant diffusion, and SMC media-to-intima migration from 1 week to 4 weeks.

Figure 1 shows the conceptual ABM flow chart that simulates CAV (Figure 1A) and the temporal details with which the cellular and molecular events occur during the 4-week timeline (Figure 1B), representing the typical period of CAV manifestation in mouse models (Pinheiro and Barros, 2009; Zhang et al., 2012; Vergani et al., 2013; Wang et al., 2021). First, within the initialization routine, the initial geometry of the ABM was generated along with the inflammatory and WSS-based stimuli. Second, the cellular and molecular events were executed, simulating CAV progression and related arterial wall remodeling until reaching the end of the proposed follow-up time ( $t=T_{sim}$ with $T_{sim}=4\;weeks$ ) or until critical occlusion of the lumen area (namely, 99% of lumen area reduction). Last, the vessel cross-section geometry was regularized to guarantee a smooth profile of the lumen border at the end of each iteration.

#### 2.1.1 Initialization

**FIGURE 2:**
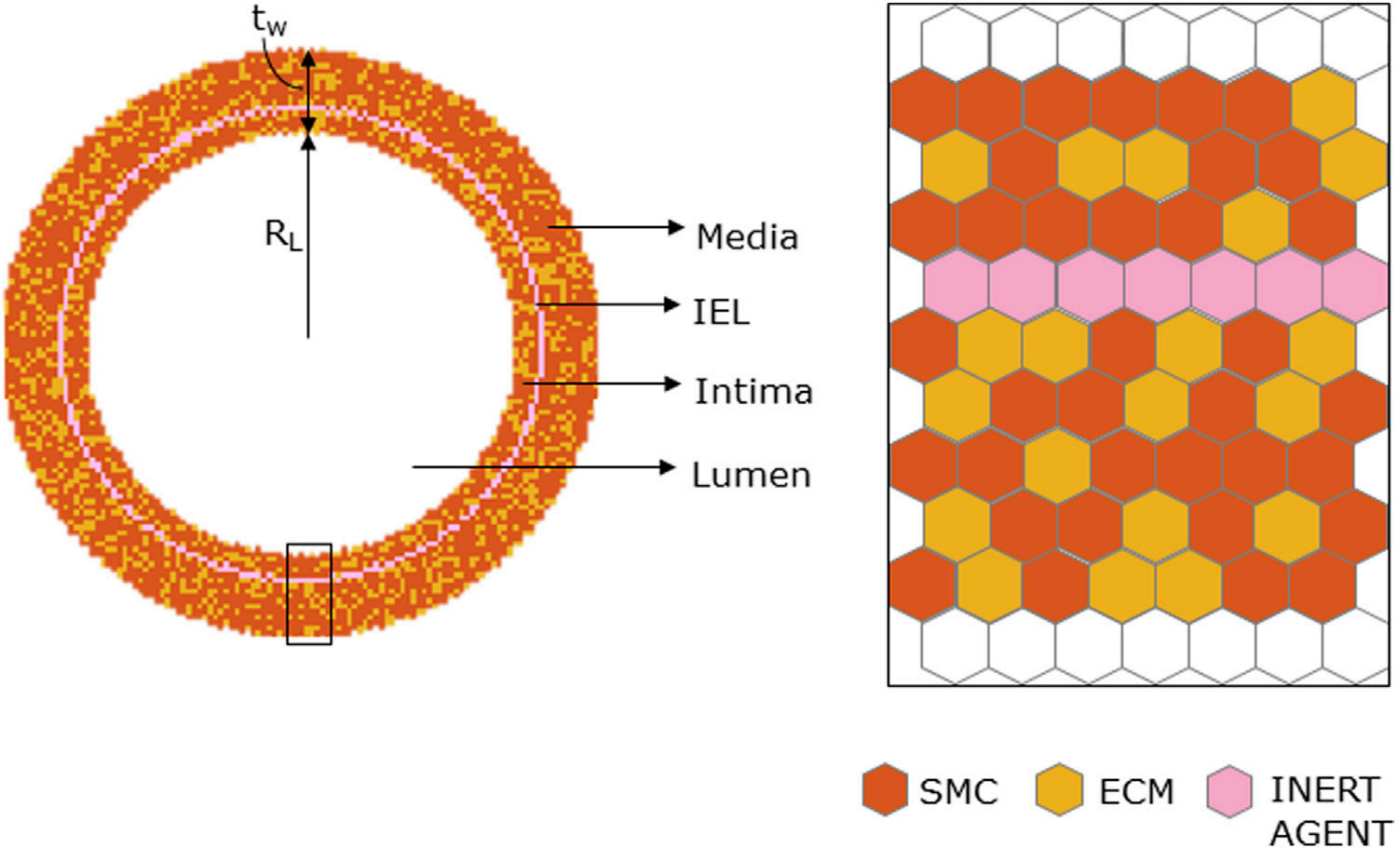
Geometrical initialization. Agent-based model cross-section with lumen, tunica intima, internal elastic lamina (IEL), and tunica media. The tunica intima and media are initially populated by smooth muscle cells (SMCs) and extracellular matrix (ECM) and IEL homogeneously filled with inert agents.

The ABM was implemented to resemble the LCA distal cross-section of a 25-week-old mouse (Pai et al., 2012). The model is laid on a 2D on-lattice <141 × 141> hexagonal grid and represents an idealized LCA cross-section with concentric lumen, intima, internal elastic lamina (IEL), and media layers (Figure 2). The artery presents a lumen radius ( $\left.R_{L}\right)$ of 80 µm and a wall thickness ( $t_{w}$ ) of 32 µm (Pai et al., 2012), which were converted in ABM sites for geometry initialization (Table 1). Specifically, a 2 µm/ABM site scaling factor was considered, thus assuming one cell per ABM site (i.e., mouse SMC diameter ~2 µm (Helms et al., 2010)). Within our ABM, we assumed that each site represents a single cell, regardless of its shape, to enhance the computational feasibility while capturing essential cellular interactions in vessel walls. Cardiac cells within coronary walls often exhibit a tapered rather than spherical morphology. To accommodate this anatomical variation, we implemented a 1/3 reduction in cell size, adjusting the cell radius from 6 µm to 2 µm. This adaptation strikes a balance between practical implementation and accurately representing cell morphology within vessel walls. The intima and media layers were randomly populated by SMC and ECM agents (60% SMC; 40% ECM), while the IEL consisted of a single layer of inert agents (Figure 2).

**TABLE 1:** Agent-based model (ABM) geometric parameters reported with name, imposed value, reference value, and literature reference.

Name	Imposed value	Reference value (µm)	References
Cell dimension	1 ABM site = 1 px	2	Helms et al. (2010)
Lumen radius—*R*_*L*_	40 sites	80	Pai et al. (2012)
Wall thickness—*t*_*w*_	16 sites	32	Pai et al. (2012)

The ABM was informed with an inflammatory input $\left(I_{input}\right.$ ) and a hemodynamic WSS-based input ( ${WSS}_{input}$ ), as detailed in Table 2. $I_{input}\in \left[0,1\right]$ was defined as a local non-symmetric (NS) stimulus within the intima layer, and it was expressed through a 2D Gaussian distribution. The formulation of ${WSS}_{input}\in \left[0,1\right]$ proposed in Corti et al. (2022b) and Corti et al. (2022a) was adopted to reproduce the increased cellular activities at low-WSS regions. Specifically, first, the lumen wall (i.e., the first layer of the tunica intima) was initialized with a WSS profile following the Poiseuille law. To initialize an NS WSS, representative of a generic curved portion of the vessel, the maximum blood velocity, $U_{\max}$ , was off-centered. Second, the variable $D\left(WSS\right)\in \left[0,1\right]$ , representing the WSS-related endothelial dysfunction, was assigned to each lumen wall site according to a sigmoid-shaped function (Corti et al., 2022a; Corti et al., 2022b). High values of $D\left(WSS\right)$ were associated to low WSS, thus reflecting the inverse relationship between WSS and synthetic/proliferative SMC condition activation. Third, to obtain a CAV triggering an input within the intima layer, ${WSS}_{input}$ was defined as a spatial decay of $D\left(WSS\right)$ within the intima layer (Corti et al., 2022a; Corti et al., 2022b). $U_{\max}$ and ${WSS}_{input}$ were updated throughout the simulation at each time step ( ∆t=1 hour) to account for the hemodynamic variation due to concurrent arterial wall remodeling, while keeping the blood flow *Q* constant.

**TABLE 2:** Agent-based model inputs.

I_input_	$I_{input}^{i}=\exp\left(\frac{{\left(x_{i}-x_{c}\right)}^{2}+{\left(y_{i}-y_{c}\right)}^{2}}{2\;\sigma }\right)$
WSS_input_	${WSS}^{i}=2\;\frac{µ\;U_{\max}\left(t\right)}{R_{I}^{i}}$
	${D\left(WSS\right)}^{i}=-\frac{1}{1+\exp\left(L_{1}\left({WSS}^{i}-L_{2}\right)+1\right)}$
	${WSS}_{input}=\left\{\begin{array}{c} {D\left(WSS\right)}^{i},if\;i\in lumen\;wall\\ \sum_{i}{D\left(WSS\right)}^{i}\;Amp\;\left(1+\cos\left(\pi \;\frac{x}{dist}\right)\right),if\;i\in intima \end{array}\right.$

$I_{input}^{i}$ , inflammatory input; (x_c_, y_c_), coordinates of the surface center arbitrary located in a random site within the top-right portion of the intima layer; *σ*, standard deviation; (x_i_, y_i_), coordinates of the evaluation intima site *i*; *WSS* ^*i*^, wall shear stress for the lumen wall site *i; µ,* blood viscosity equal to 3.5 cP (Panteleev et al., 2021); *U*_*max*_, maximum blood velocity; $R_{I}^{i}$ , distance between the lumen wall site *i* and the location of $U_{\max}$ . *D*(*WSS*), level of endothelial dysfunction for the lumen wall site *i*; *L1*, sigmoid curve slope; *L2*, WSS value at which *D(WSS)* = 0.5; *WSSinput*, WSS-based input; *Amp*, cosine function amplitude; *x*, distance between the intima site (evaluation site of *WSSinput*) and the lumen wall site *i,* with x < dist ; dist, 20 constant.

#### 2.1.2 Cellular and molecular events

Cellular/molecular events were governed by probabilistic rules and were set to occur with a specific timing throughout the simulation. SMC/MP mitosis and apoptosis and ECM production/degradation were marked by the related agent-specific cycle. At t=0 , an internal clock $T_{clock}$ was assigned to each agent spanning between 0 and $T_{agent}$ (the latter being representative of the agent biological state), with $T_{agent}=T_{SMC}=12\;hours$ for SMCs, $T_{agent}=T_{ECM}=2\;hours$ for ECM, and $T_{agent}=T_{MP}=12\;hours$ for MPs (Garbey et al., 2017). Specifically, $T_{clock}$ indicates the temporal instant of the cell cycle that a specific cell is currently at, hence representing the cell progression throughout the cell cycle. During the initialization phase of our model, $T_{clock}$ is randomized for each individual cell. This randomness ensures that cells are not synchronized at the start, emulating the natural diversity in cell cycle stages that exist within real biological systems. $T_{SMC}$ , $T_{ECM}$ , and $T_{MP}$ represent the duration of the cycle for SCM (12 h), ECM (2 h), and MP (12 h), respectively. The $T_{clock}\in \left[0,T_{agent}\right]$ of each agent was incremented by *1 h* at every simulation step and once $T_{clock}=T_{agent}$ , the agent became potentially active (i.e., the related event could occur according to the stochasticity of the simulation). At this point, the potentially active agents were randomly investigated with a Monte Carlo method to determine event occurrence. Specifically, a randomly CPU-generated number $test\in \left[0,1\right]$ (two significant digits) was compared with the agent-specific event probability, and the event occurred if test < $p_{event}$ . Once the event occurrence was verified, the arterial wall configuration was updated to account for agent generation (in case of cell mitosis or ECM production) or agent removal (in case of cell apoptosis or ECM degradation). Specifically, inward- and outward-oriented tissue organizations were implemented in the intima and media, respectively, as in Corti et al. (2020). As shown in Supplementary Figures S1, S2, according to the inward- and outward-oriented processes, agent generation/removal in the intima determines intimal growth/shrinking with lumen area variation, while agent generation/removal in the media determines media growth/shrinking without lumen area variation.

MP infiltration and SMC media-to-intima migration occurred for t belonging to the specific time window illustrated in Figure 1B, and at each time step, the event occurrence was tested by a Monte Carlo method, as explained previously. For these events, details on the tissue reorganization are provided in Section 2.1.2.1 and Section 2.1.2.3.

The probability rules defined for each event and the corresponding parameter values are listed in Table 3 and Table 4, respectively, and detailed as follows. For the parameters included in the sensitivity analysis, their range, rather than a value, is provided, and the explanation is detailed in Section 2.2.

**TABLE 3:** Agent-based model probability equations.

MP activity	
Infiltration	$P_{MP\;infiltration}^{i}=\alpha_{7}\;\left(1+I_{input}^{i}\right)\;\left(1+\alpha_{8}∙\exp\left(-{dist}_{MP}^{i}\right)\right)$ $\left(1+\frac{{MP}_{group}}{\alpha_{9}}\;\right)$ $t_{i}\leq t\leq t_{c}$
Dynamics	$\begin{array}{c} \left\{\begin{array}{c} P_{{MP}_{mitosis}}=\alpha_{10}\\ P_{{MP}_{apoptosis}}=0.5*\alpha_{10} \end{array}\right.\;t_{i}<t\leq t_{c} \end{array}$
	$\left\{\begin{array}{c} P_{{MP}_{mitosis}}={0.5∙\alpha }_{10}\\ P_{{MP}_{apoptosis}}=\alpha_{10} \end{array}\right.\;t>t_{c}$
Chemoattractant diffusion	$Ca\left(x,y,t_{d}\right)=\frac{{Ca}_{0}}{\sqrt{4\;\pi \;D\;t_{d}}}\;e^{-\frac{\left(x^{2}+y^{2}\right)}{4\;D\;t_{d}}}$
	${Ca}_{norm}^{i}\left(t\right)=\frac{{Ca}^{i}\left(t\right)-{Ca}_{\min}}{{Ca}_{\max}-{Ca}_{\min}}$
SMC dynamics	
Intima	$\left\{\begin{array}{c} p_{SMC\;mitosis}^{i}=\alpha_{1}+\alpha_{3}∙I_{input}^{i}+\alpha_{4}∙n_{MP}+\alpha_{11}∙{WSS}_{input}^{i}+\alpha_{13}∙{Ca}_{norm}^{i.}\\ p_{SMC\;apoptosis}^{i}=\alpha_{1} \end{array}\right.$
Media	$\left\{\begin{array}{c} p_{SMC\;mitosis}^{i}=\alpha_{1}\\ p_{SMC\;apoptosis}^{i}=\alpha_{1} \end{array}\right.$
ECM dynamics	
Intima	$\left\{\begin{array}{c} p_{ECM\;production}^{i}=\alpha_{2}+\alpha_{5}∙I_{input}^{i}+\alpha_{6}∙n_{MP}+\alpha_{12}∙{WSS}_{input}^{i}\;{+\alpha }_{14}∙{Ca}_{norm}^{i}\\ p_{ECM\;degradation}^{i}=\gamma ∙\alpha_{2} \end{array}\right.$
Media	$\left\{\begin{array}{c} p_{ECM\;production}^{i}=\alpha_{2}\\ p_{ECM\;degradation}^{i}=\gamma ∙\alpha_{2} \end{array}\right.$

$P_{MP\_infiltration}^{i}$ , probability of MP infiltration; $t_{i}$ , infiltration starting time; $t_{c}$ , infiltration peak time; $P_{{MP}_{mitosis}}$ , probability of MP mitosis; $P_{{MP}_{apoptosis}}:$ , probability of MP apoptosis; $Ca\left(x,y,t_{d}\right)$ , concentration of the chemoattractant at given spatial coordinates and time; $t_{d}\in \left[t_{0},t_{D}\right]$ , time pointer; $t_{D}$ , total time of the chemoattractant diffusion; $t_{D}=T_{sim}{-t}_{c}=3weeks$ , *Ca*_0_ initial chemoattractant concentration at the MP cluster centroid coordinates; *D*, diffusion coefficient; ${Ca}_{norm}^{i}\left(t\right)$ , normalized chemoattractant concentration for site *i* at given time; ${Ca}_{\min}$ and ${Ca}_{\max}$ , minimum and maximum of *Ca* range, respectively; $p_{SMC\;mitosis}^{i}$ , probability of SMC mitosis; $p_{SMC\;apoptosis}^{i}$ , probability of SMC apoptosis; $p_{ECM\;production}^{i}$ , probability of ECM production; $p_{ECM\;degradation}^{i}$ , probability of ECM degradation; $I_{input}^{i}$ , inflammatory input; $n_{MP}:$ , number of MP neighbors surrounding site *i*; ${WSS}_{input}^{i}$ , WSS-based input for site *i*.

**TABLE 4:** Agent-based model (ABM) parameters.

Parameter	Description	Value/range
α_1_	Baseline SMC mitosis and apoptosis	0.014
α_2_	Baseline ECM production	0.006
γ	Calibrated baseline coefficient to counterbalance tendency in ECM production	1.301
α_3_	Effect of the *I*_*input*_ on SMC mitosis	(0; 0.034)
α_4_	Influence of the neighboring MP on SMC mitosis	(0; 0.0056)
α_5_	Effect of the *I*_*input*_ on ECM production	(0; 0.036)
α_6_	Influence of the neighboring MP on ECM production	(0; 0.006)
α_7_	MP intrinsic probability of infiltration	(0; 0.019)
α_8_	Weights of the distance term between *i*th site and its closest MP	(0; 1.65)
α_9_	Normalization constant of the MP group	(6; 60)
α_10_	MP dynamics	(0; 0.15)
α_11_	Influence of *WSS*_*input*_ on SMC mitosis	(0; 0.034)
α_12_	Influence of *WSS*_*input*_ on ECM production	(0; 0.036)
α_13_	Influence of *Ca*_*norm*_ on SMC mitosis	(0; 0.034)
α_14_	Influence of *Ca*_*norm*_ on ECM production	(0; 0.036)
L_1_	Slope of *D(WSS)*	(−3;−0.28)
L_2_	Half decay of *D(WSS)*	(8; 12)
Amp	Amplitude of the cosine function representing the diffusion of *D(WSS)* in the intima	(0.01; 0.10)
try	Number of possibilities for MPs to infiltrate	(10; 100)
C_radius_	Radius of chemoattractant action for SMC migration	(4.55; 56)

$I_{input}$ , inflammatory input; ${WSS}_{input}$ , wall shear stress input; SMC, smooth muscle cell; ECM, extracellular matrix; MP, macrophage; ${Ca}_{norm}$ , normalized chemoattractant concentration; $D\left(WSS\right)$ , variable representing the level of endothelial dysfunction.

##### 2.1.2.1 MP activities

**FIGURE 3:**
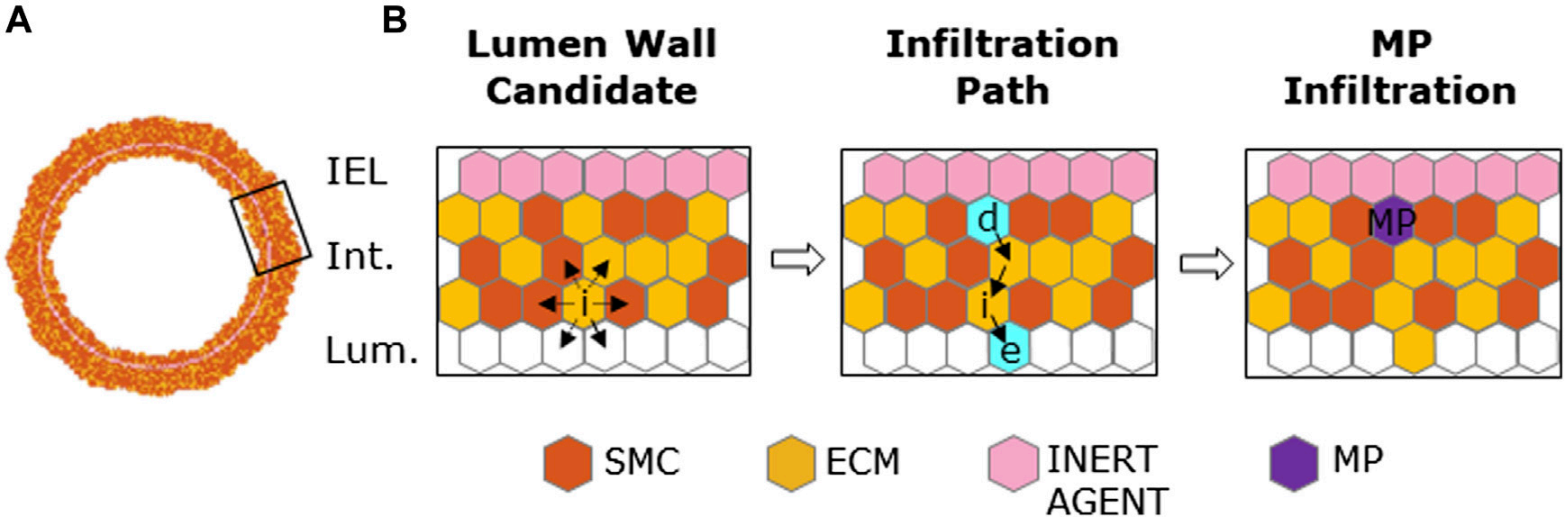
Macrophage (MP) infiltration sequence. **(A)** Artery cross-section at MP infiltration with its cellular components: smooth muscle cells (SMCs), extracellular matrix (ECM), inert agent, and MPs. **(B)** Left panel: active site (i) explores the content of neighbors and targets an empty site in the lumen and an occupied one in the intima. Central panel: site (e) and (d) (in cyan) are the designated sites in the lumen and in the intima, respectively, with solid black arrows explaining shifting movement among the wall. Right panel: site (d) filled with an MP agent (in purple).

MP infiltration and dynamics were activated starting from $t\geq t_{i}$ with $t_{i}=2\;days$ , the timing at which usually graft-infiltrating cells are observed (McLean et al., 1997). MP infiltration was governed by the probability of a lumen wall site to allow MP extravasation and access to the intimal compartment (Corti et al., 2020), detailed in Table 3 with parameter range and definition provided in Table 4. The probability of MP infiltration $P_{MP\;infiltration}^{i}$ was set to promote MP infiltration in areas of i) high $I_{input}^{i}$ and ii) already presenting intimal MPs (by considering both the distance and number of MPs). At each time step, only one MP invades the intima. A number $n={try}_{MP}$ of lumen sites with the highest $P_{MP\;infiltration}^{i}$ were considered candidate access sites and explored in descending order of $P_{MP\;infiltration}^{i}$ . Then, if $test\;<P_{MP\;infiltration}^{i}$ , the site *i* was marked as an MP access point to the intima; otherwise, the following site of the list was investigated up to exhausting the ${try}_{MP}$ possibilities. Once the access site was selected, the MPs infiltrated and the intima remodeled, as shown in Figure 3. With *i* being the access site, an empty neighboring site *e* and a deeper intimal site *d* were randomly selected so that the content of the *i* site was shifted in *e*, the agent in *d* was moved in *i,* and finally *d* was filled with an MP agent.

A two-phase MP mitosis/apoptosis was implemented to qualitatively replicate the dynamic trend of the graft-infiltrating cells (e.g., MPs, monocytic and lymphocytic cells) in the first days after organ transplantation (McLean et al., 1997). Specifically, the mitosis/apoptosis probability rules were defined so that i) MP mitosis was promoted within the range $t_{i}<t\leq t_{c}$ , with $t_{c}=1\;week$ corresponding to the time when the maximum peak of graft-infiltrating cells was observed (McLean et al., 1997; Ordikhani et al., 2020) and ii) MP apoptosis was favored for $t>\;t_{c}$ . Tables 3 and Table 4 list the probabilities of MP mitosis and apoptosis ( $P_{{MP}_{mitosis}}$ and $P_{{MP}_{apoptosis}})$ and the parameters, respectively.

Once the MP cluster formed and reached the highest number of cells (t = $t_{c}$ ), the MP cluster started releasing a generic chemoattractant, which enhanced SMC proliferation and ECM production and promoted SMC media-to-intima migration (Kloc and Ghobrial, 2014; Von Rossum et al., 2014). The diffusion of chemoattractant concentration $\left(Ca\right.$ ) was computed according to a Gaussian distribution law detailed in Table 3. Similarly to what was done for the tumor growth factor diffusion in Araujo et al. (2014), the diffusion coefficient D= 900 μm^2^/min (Goodhill, 1998) was converted to $D=0.45\;px/t_{D}$ , by normalizing with respect to the total time $t_{D}$ in which the phenomenon occurs. Ca was normalized $\left({Ca}_{norm}\right)$ between 0 and 1 as shown in Table 3, to cope with the range of the probability laws.

##### 2.1.2.2 SMC and ECM dynamics

Under baseline conditions, namely, in the absence of perturbations, balanced SMC mitosis/apoptosis and ECM production/degradation were set to guarantee a physiologic arterial wall remodeling (Garbey et al., 2015; Garbey et al., 2017; Corti et al., 2019; Corti et al., 2020). In the presence of $I_{input}$ and ${WSS}_{input}$ , SMC mitosis and ECM production in the intima were perturbed. The probabilities $p_{SMC\;mitosis}^{i}$ , $p_{SMC\;apoptosis}^{i}$ , $p_{ECM\;production}^{i}$ , and $p_{ECM\;degradation}^{i}$ are detailed in Table 3, and the parameter values and ranges are provided in Table 4. The baseline parameters (i.e., α1 and α2) were established by initializing initial estimates and retrieving values consistent with the findings of Garbey et al. (2017) and Corti et al. (2019, 2020). To ensure that the model aligns with empirical observations, an essential aspect was the assignment of values for both ECM production and degradation. Given that SMCs contribute to ECM production, the challenge emerged in assigning a single value for both processes. An equal probability allocation would have led to elevated ECM production due to the slightly greater number of SMC components during model initialization (60% SMC; 40% ECM). As a solution, we introduced a coefficient γ, which was calibrated to determine the ECM degradation probability. To calibrate γ, we explored five γ values denoted as γ ⃗ = {1, 1.2, 1.275, 1.345, and 1.5}. For each potential γ value, we conducted N = 10 simulations. Calibration was performed through two main steps: first, by comparing the final versus initial ECM ratio, averaged over the ten simulations for each γ value and second, by interpolating the ECM ratio against γ when the ECM ratio equaled 1.

##### 2.1.2.3 SMC media-to-intima migration

**FIGURE 4:**
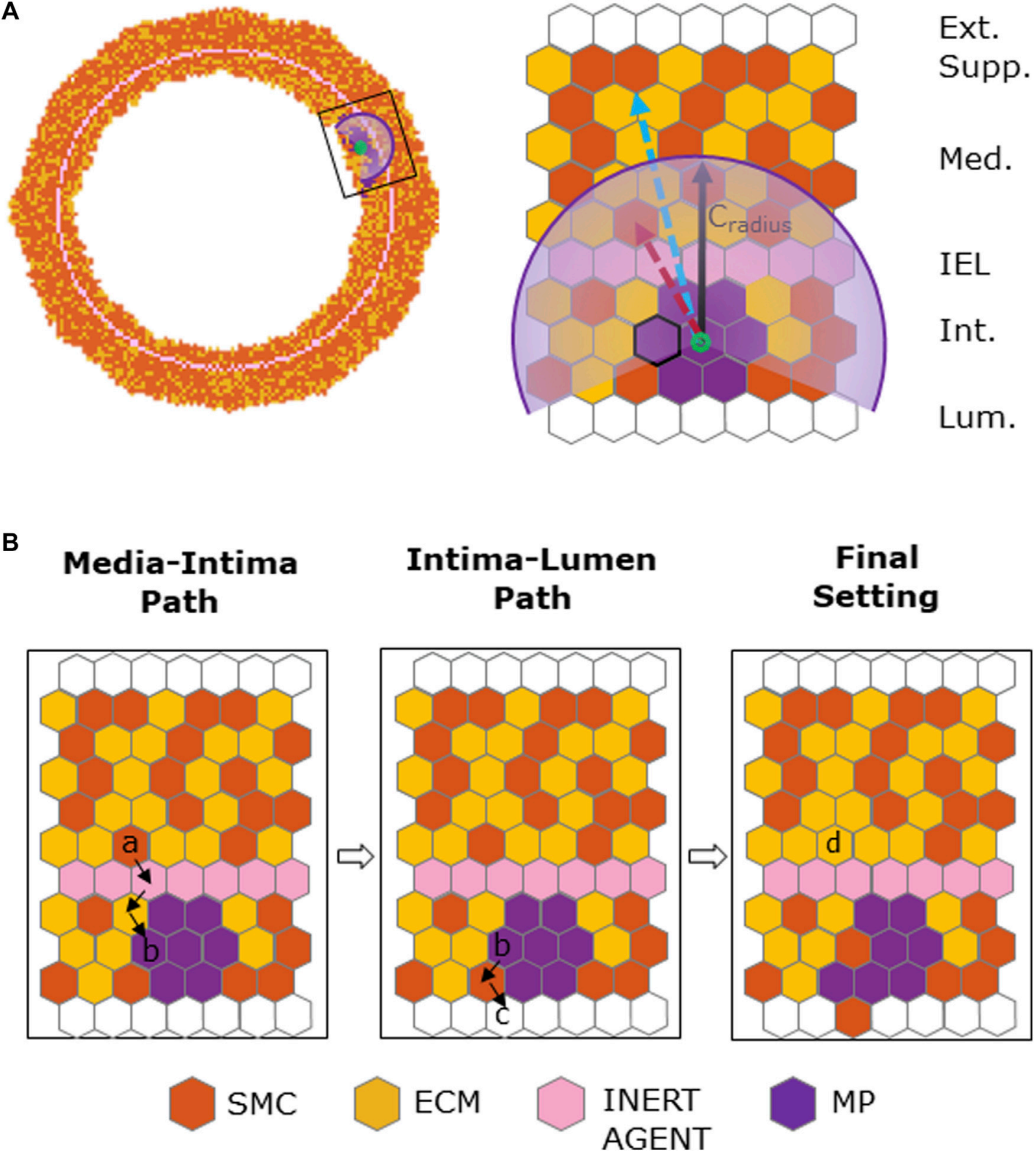
Chemoattractant-induced SMC media-to-intima migration. **(A)** Coronary cross-section at $t=t_{c}$ with magnification showing a circular section of the chemoattractant distribution from the MP cluster centroid (in green). In the magnified ABM grid, two types of SMC media sites are observed: the red dashed arrow indicates a potential candidate for media-to-intima migration, while the blue dashed arrow indicates an excluded candidate site for migration. The intima site with the marked edge is a potential end-site for the media-to-intima migration path. **(B)** SMC media-to-intima migration process. From left to right: media–intima path involving the chosen media SMC starting site (a) and intima ending site (b); intima–lumen path involving the intima starting site (b) (i.e., the ending site of the media–intima path) and the ending lumen site (c); the final setting showing an inward-remodeling effect with a new media ECM site (d) taking the place of the migrated SMC.

SMC migration occurred when $t\geq t_{c}$ , with a rate of 1 migrating SMC/h (Waters et al., 2011). Figure 4A outlines the implemented stochastic cell migration process, in which i) the potential medial migrating SMC was randomly selected among the cells lying within the critical radius, $C_{radius}$ (originating from the MP cluster centroid), of the ${Ca}_{norm}$ region of influence and ii) the potential intimal end-site was randomly selected among the sites (i.e., SMC/ECM/MP site) with ${Ca}_{norm}\neq 0$ . The migration occurs if ${{test}_{migr}<\;Ca}_{norm}^{i}$ , the former being a random CPU-generated number. SMC media-to-intima migration implies that the SMC migrates to the elected site, which was freed through an inward-oriented movement of the surrounding elements, while an ECM element is placed in the original SMC site to fill the void, as depicted in Figure 4B. This leads to a progressive reduction of SMC content in the media in favor of the ECM (Kloc and Ghobrial, 2014). MP and IEL were assumed not to represent an obstacle in the migration process. Indeed, experimental evidence showed that migrating SMCs register an increase of the viscosity, becoming less adhesive to their neighbors, and thus being able to pass more easily through the wall tissue (Louis and Zahradka, 2010). Overall, SMC media-to-intima migration resulted in intimal thickening and lumen narrowing.

#### 2.1.3 Geometry regularization

To guarantee structure integrity and a smooth inner and outer profile, IEL and border regularizations were applied after SMC/ECM dynamics and SMC media-to-intima migration, respectively. Specifically, at the inner and outer borders, agents were locally redistributed to maximize their contact as conducted in previous works (Garbey et al., 2017; Corti et al., 2019; Corti et al., 2020; Corti et al., 2022a; Corti et al., 2022b), while the IEL was regularized to re-establish the initial configuration after SMC migration as detailed in Supplementary Figure S3.

### 2.2 Sensitivity analysis

A sensitivity analysis was performed to quantify the impact of the set of *j* = 17 ABM parameters $\left\{\alpha_{3},\alpha_{4},\alpha_{5},\alpha_{6},\alpha_{7},\alpha_{8},\alpha_{9},\alpha_{10},\alpha_{11},\alpha_{12},\alpha_{13},\alpha_{14},L_{1},L_{2},Amp,try,c_{radius}\right\}$ on the ABM outputs at 1-week and 4-week follow-ups. For the parameters included in the sensitivity analysis, their range (Table 4) was defined as detailed as follows. As previously proposed by Corti et al. (2022b), the ranges of *L*_1_ and *L*_2_ were set to satisfy a 10% tolerance on the value of D(WSS), meaning that $D\left(WSS=0\right)>0.9$ and $D\left(WSS=20\right)<0.1$ . The range of *Amp* was defined to account for extreme cases of minimum and maximum propagation of ${WSS}_{input}$ in the intima. The ranges of $\alpha_{3},\alpha_{4},\alpha_{5},\alpha_{6},\alpha_{7},\alpha_{8},\alpha_{9},\alpha_{10},\alpha_{11},\alpha_{12},\alpha_{13,}$ and $\alpha_{14}$ were defined by running worst-case scenarios (i.e., by setting all the other parameters to the most CAV-prone values) and selecting as upper limits values that guarantee an admissible event probability ( p≤1 ) and preventing the total occlusion before the 4-week follow-up. The range of $c_{radius}$ was defined by setting the external edge radius as the maximum value (i.e., 56 sites in the grid units) and as the minimum value the minimum distance between the MP cluster centroid and the first layer of the media obtained among 10 repeated simulations (each leading to a different MP cluster centroid). Finally, the range of try was defined as tenfold the range used in Corti et al. (2020), to be consistent with the range progression of the other parameters.

Latin hypercube sampling (LHS) was adopted to sample the admissible range of the *j* parameters in k=2000 equal intervals, with *k* chosen to have a *k/j* proportion consistent with the work by Corti et al. (2022b), which guaranteed statistical significance in the sensitivity analysis. Consequently, an LHS matrix ( k×j ) was obtained as proposed in Corti et al. (2020, 2022b), and for each *k* parameter combination, three repetitions were run, leading to 6,000 total simulations. Partial rank correlation coefficients (PRCCs) were computed in MATLAB between each ABM parameter and the outputs of interest at the 1-week and 4-week follow-ups, namely, the lumen and intimal areas and the intimal content of SMCs, ECM, and MPs (Marino et al., 2008; Corti et al., 2020; Corti et al., 2022b). To compute the PRCCs, the average ABM outputs obtained from the three repetitions were considered. Statistically significant correlations were assumed for *p*-value <0.05 (with false discovery rate correction).

### 2.3 Definition of scenarios

**FIGURE 5:**
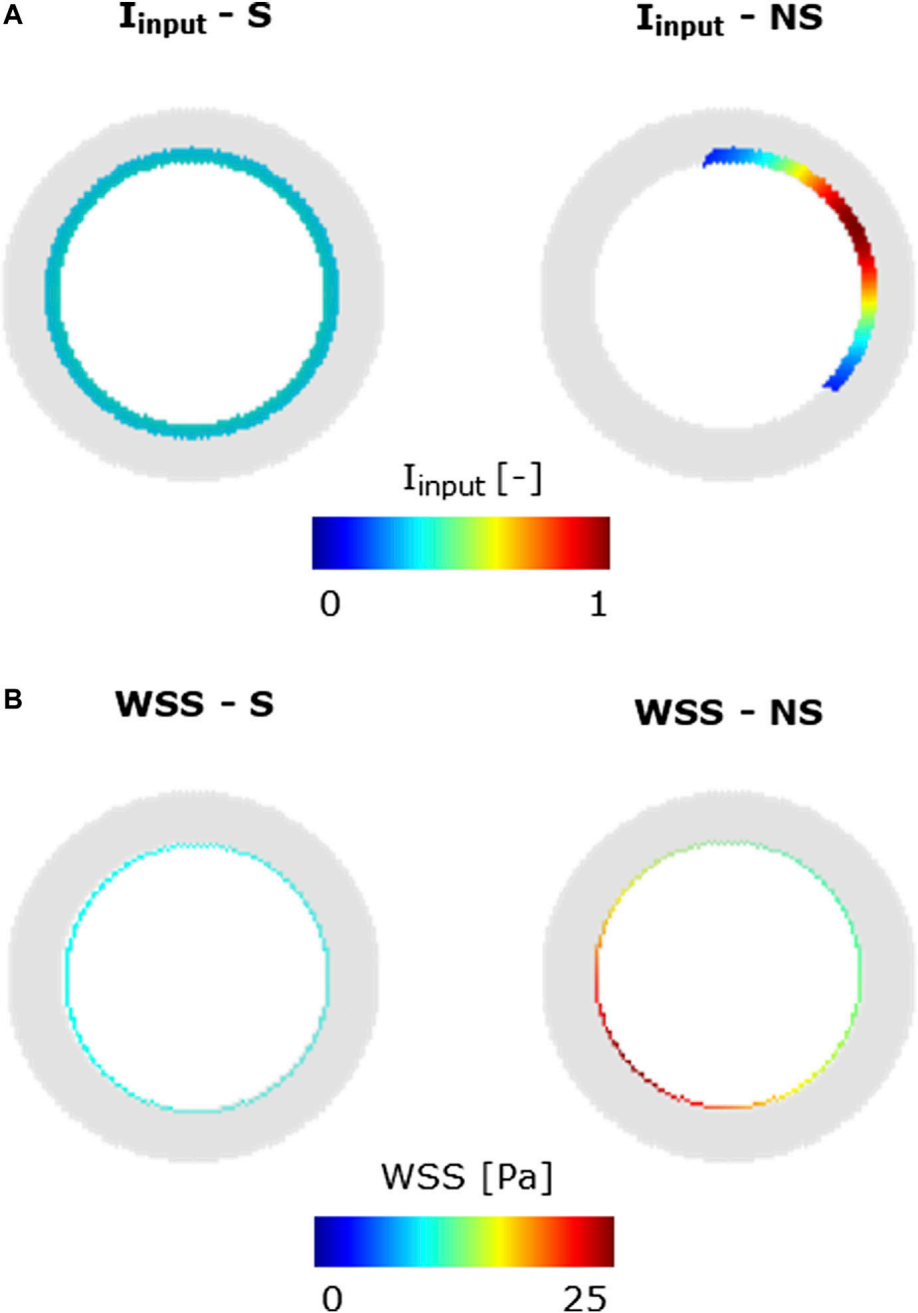
Inflammatory/hemodynamic-based input stimuli. **(A)** $I_{input}$ conditions: symmetric (S) and non-symmetric (NS). **(B)** WSS conditions: symmetric (S) and non-symmetric (NS).

The ABM response to different conditions was investigated by setting the ABM parameters presented in Table 4 at their half-range values. Fourteen scenarios (detailed in Table 5) were generated and were characterized by the combination of different inflammatory and hemodynamic WSS-based inputs shown in Figure 5 and by the activation/deactivation of MP infiltration/dynamics and chemoattractant diffusion events.

**TABLE 5:** Agent-based model testing scenarios 1–14.

Scenario	1	2	3	4	5	6	7	8	9	10	11	12	13	14
$I_{input}$	S-0.5	Abs	NS	Abs	S-0.5	NS	S-0.5	S-0.5	NS	NS	S-0.5	S-0.5	NS	NS
${WSS}_{input}$	Abs	S-0.5	Abs	NS	S-0.5	NS	Abs	Abs	Abs	Abs	S-0.5	S-0.5	NS	NS
**MP**	Abs	Abs	Abs	Abs	Abs	Abs	Prs	Prs	Prs	Prs	Prs	Prs	Prs	Prs
Ca	Abs	Abs	Abs	Abs	Abs	Abs	Abs	Prs	Abs	Prs	Abs	Prs	Abs	Prs

$I_{input}$ , inflammatory input; ${WSS}_{input}$ , wall shear stress input; MP, macrophage; Ca , chemoattractant; S, symmetric; NS, non-symmetric; Abs, absent; Prs, present.

Specifically, in scenarios 1–4, only one stimulus, either inflammatory or hemodynamic, was considered, with the MP and chemoattractant events deactivated. In scenarios 5 and 6, both the inflammation and hemodynamic stimuli were present, with the MP and chemoattractant events deactivated. Finally, in scenarios 7–14, the MP and chemoattractant events were activated and combined with different inflammation and hemodynamic WSS-based inputs. As regards the inflammation input, i) a S and uniform inflammation equal to 0.5 (representative of a mid-level inflammation) and ii) a NS inflammation localized at the top-right portion of the cross-section, with a peak of 1, were generated. As regards the hemodynamic input, i) an S WSS profile with WSS ~ 10 Pa (representative of a mid WSS value) and ii) NS WSS with low WSS $\left(WSS\;<\;10\;Pa\right)$ localized at the top-right portion of the cross-section were generated, by imposing a flow-rate Q=0.13 ml/min (Teng et al., 2016; Feng et al., 2018) with centered peak velocity and a flow-rate Q=0.20 ml/min (Teng et al., 2016; Feng et al., 2018) with off-centered peak velocity, respectively.

### 2.4 Generation of CAV cases

**FIGURE 6:**
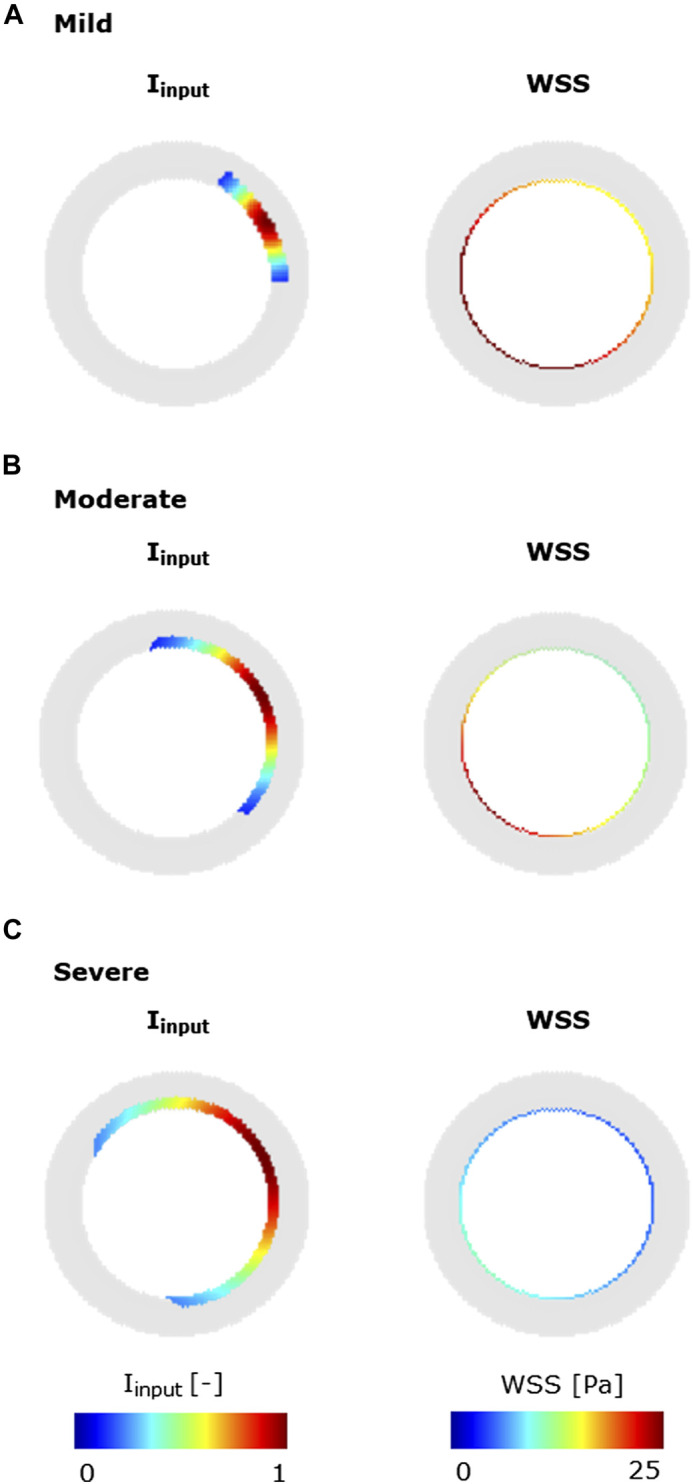
Inflammatory/hemodynamic-based stimuli leading to characteristic CAV manifestations. **(A)** Mild-CAV model initialized with a small inflammation region and WSS profile with a minimum value of 15 Pa and a maximum value of 35 Pa. **(B)** Moderate-CAV model initialized with the mid-dimension inflammation region and WSS profile with a minimum value of 8 Pa and a maximum value of 25 Pa. **(C)** Severe-CAV model initialized with a large inflammation region and WSS profile with a minimum value of 5 Pa and a maximum value of 10 Pa. The displayed WSS range was set to [0 25] Pa.

To explore the heterogeneous response of the ABM to different levels of $I_{input}$ and ${WSS}_{input}$ (mild, moderate, and severe), two further cases were generated starting from the previously introduced scenario 14 (Table 5), representing the most general situation, i.e., NS inputs, and activated MP activities (i.e., MP infiltration, dynamics, and chemoattractant diffusion). By considering the inputs defined in scenario 14, moderate-, mild-, and severe-input cases were generated (Figure 6). As regards $I_{input}$ , the mild, moderate, and severe cases were defined by varying the region of exposure to the input. As regards ${WSS}_{input}$ , mild, moderate, and severe cases were obtained by imposing Q=0.30 mL/min, Q=0.20 ml/min, and Q=10 ml/min (Teng et al., 2016; Feng et al., 2018) with off-centered peak velocity, respectively. Both $I_{input}$ and the WSS profile were defined such that the CAV trigger was localized in the top-right portion of the cross-section.

## 3 Results

### 3.1 Findings of sensitivity analysis

**FIGURE 7:**
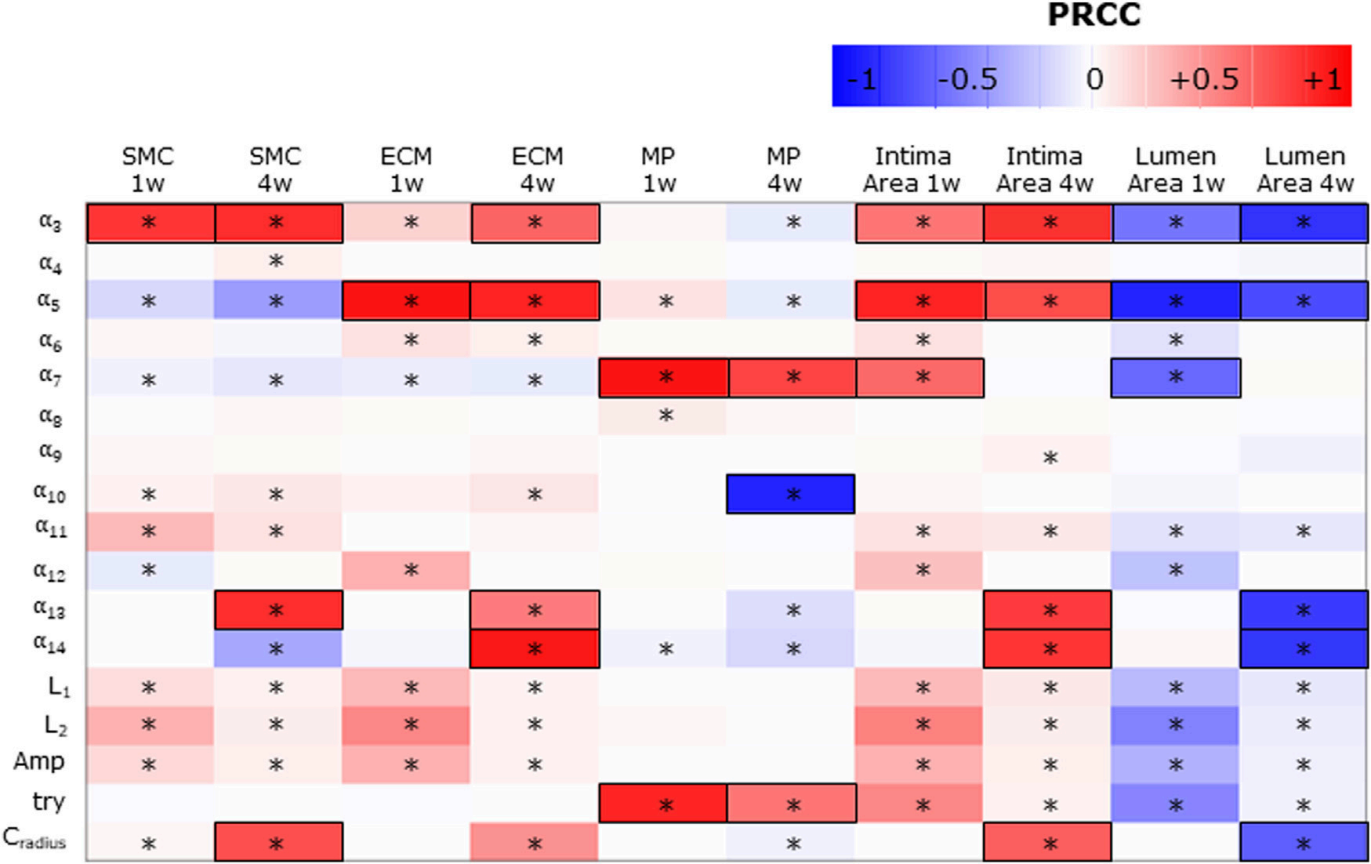
Sensitivity analysis results for intima variables, intima, and lumen areas. Partial rank correlation coefficients (PRCCs) between the ABM parameters and 1-week and 4-week smooth muscle cell (SMC), extracellular matrix (ECM), macrophage (MP), intima, and lumen areas. PRCCs range from −1 (blue) to +1 (red), with negative PRCCs corresponding to a negative correlation and positive PRCCs to a positive correlation. |PRCC| > 0.5 is squared in black. (*) Significant PRCC, *p-*value <0.05.

Figure 7 shows the PRCCs between the *j* = 17 parameters and the five ABM outputs of 17 ABM sites, at two time points, i.e., at the 1-week and 4-week follow-ups. At 1 week, four out of 17 parameters (i.e., $\alpha_{3},\alpha_{5},\alpha_{7},try$ ) presented significant PRCCs for at least three out of five investigated outputs and $\left|PRCC\right|\;>\;0.5$ for at least one out of five. Specifically, α_3_ exhibited statistical significance (p < 0.05) across the SMC and ECM contents, intima, and lumen areas, yielding a |*PRCC*| value of 0.78 for the SMC content and 0.53 for both the intima and lumen areas. Moreover, α_5_ demonstrated statistical significance (p < 0.05) across the SMC, ECM, and MP contents, as well as the intima and lumen areas, yielding a |*PRCC*| value of 0.91 for the ECM content and 0.85 for both the intima and lumen areas. Similarly, α_7_ exhibited statistical significance (p < 0.05) across the SMC, ECM, and MP contents, intima, and lumen areas, with a |*PRCC*| value of 0.91 for the MP content and 0.58 for both the intima and lumen areas. At 4 week, seven out of 17 parameters (i.e., $\alpha_{3},\alpha_{5},\alpha_{7},\alpha_{10},\alpha_{13},\alpha_{14}$ and $c_{radius}$ ) met the same criteria. Notably, α_3_ exhibited statistical significance (p < 0.05) across the SMC, ECM, and MP contents, as well as the intima and lumen areas, with a |*PRCC*| value of 0.82 for the SMC content, 0.60 for the ECM content, and 0.80 for both the intima and lumen areas. Similarly, α_5_ demonstrated statistical significance (p < 0.05) in the SMC, ECM, and MP contents, intima, and lumen areas, with a |*PRCC*| value of 0.85 for the ECM content and 0.70 for both the intima and lumen areas. α_7_ revealed statistical significance (p < 0.05) in SMC, ECM, and MP contents, with a |*PRCC*| value of 0.72 for MP content. α_10_ also showed statistical significance (*p* < 0.05) in SMC, ECM, and MP contents, with a |*PRCC*| value of 0.85 for MP content. Moreover, α13 exhibited statistical significance (*p* < 0.05) across the SMC, ECM, and MP contents, intima, and lumen areas, with a |*PRCC*| value of 0.81 for SMC content and 0.76 for both the lumen and intima areas. Similarly, α_14_ demonstrated statistical significance (*p* < 0.05) in the SMC, ECM, and MP contents, intima, and lumen areas, with a |*PRCC*| value of 0.90 for ECM content and 0.78 for both the lumen and intima areas. Finally, *C*_*radius*_ displayed statistical significance (*p* < 0.05) across the SMC, ECM, and MP contents, intima, and lumen areas, with a |*PRCC*| value of 0.70 for the SMC content and 0.62 for both the lumen and intima areas.

Consequently, $\alpha_{3},\alpha_{5},\alpha_{7},\alpha_{10},\alpha_{13},\alpha_{14},try$ and $c_{radius}$ were identified as the driving ABM parameters. Overall, the ABM response was governed by the inflammatory-related events rather than the WSS-related ones. Indeed, the WSS-related parameters (i.e., $\alpha_{11},\alpha_{12},L_{1},L_{2}$ and Amp ) presented some significant but very low correlations with all the outputs of interest both at the 1-week and 4-week follow-ups ( $\left|PRCC\right|\;<\;0.5$ ).

SMC and ECM intima contents are determined by SMC mitosis/apoptosis and media-to-intima migration and ECM production/degradation. As regards SMC mitosis/apoptosis and ECM production/degradation, the parameters associated with $I_{input}$ ( $\alpha_{3}$ for SMC and $\alpha_{5}$ for ECM) and ${Ca}_{norm}$ ( $\alpha_{13}$ for SMC and $\alpha_{14}$ for ECM) were positively and highly correlated with the corresponding intima variables, with $\alpha_{13}$ and $\alpha_{14}$ activating later, thus being relevant only at 4 weeks. Moreover, higher values of $\alpha_{3}$ and $\alpha_{13}$ directly driving SMC mitosis led not only to increased SMC content but also to increased ECM content since SMC directly mediates ECM production. Differently, $\alpha_{5}$ and $\alpha_{14}$ , driving ECM production, presented a negative correlation with SMC content, underlining the apparently counterintuitive behavior of the ABM: an increase in a parameter that promotes one cellular event (ECM production in this case), leading to the reduction of a competitive cellular event (SMC proliferation in this case). As regards SMC media-to-intima migration, as expected, $c_{radius}$ , identifying the chemoattractant spectrum of action ${Ca}_{norm}$ on the SMC media-to-intima migration, was positively correlated with the intima SMC content. Again, ECM being deposited by SMCs, an increase in SMC corresponds to an increase in ECM and, consequently, a positive correlation was found between $c_{radius}$ and the intima ECM. Notably, although being a major event in the CAV process, the presence of surrounding MP did not significantly drive SMC and ECM contents (i.e., $\alpha_{4}$ and $\alpha_{6}$ did not present significant PRCCs).

The MP intima content is determined by MP infiltration and MP mitosis/apoptosis. As regards the parameters driving MP infiltration, $\alpha_{7}$ , which is associated with $I_{input}$ , and try *,* representing the chances of MP infiltration, presented significant and high positive PRCCs with the intima content at the 1-week and 4-week follow-ups. Instead, $\alpha_{10}$ , driving MP mitosis/apoptosis, did not present significant PRCC at 1-week follow-up, but was significant and highly negative correlated with the MP intima content at 4 weeks, coherently with the timing of MP dynamics and the major role of $\alpha_{10}$ in MP apoptosis at later times. $C_{radius}$ was negatively correlated with MP content, thus confirming the counterintuitive tendency of the ABM, as previously noted.

Finally, the parameters promoting the cellular contents (i.e., $\alpha_{3}$ , $\alpha_{13}$ , and $c_{radius}$ in SMC; $\alpha_{3}$ , $\alpha_{5}$ , $\alpha_{12}$ , and $c_{radius}$ in ECM; and $\alpha_{7},try$ in MP) presented significative positive correlations with the intima area and negative ones with the lumen area.

### 3.2 Test scenarios

**FIGURE 8:**
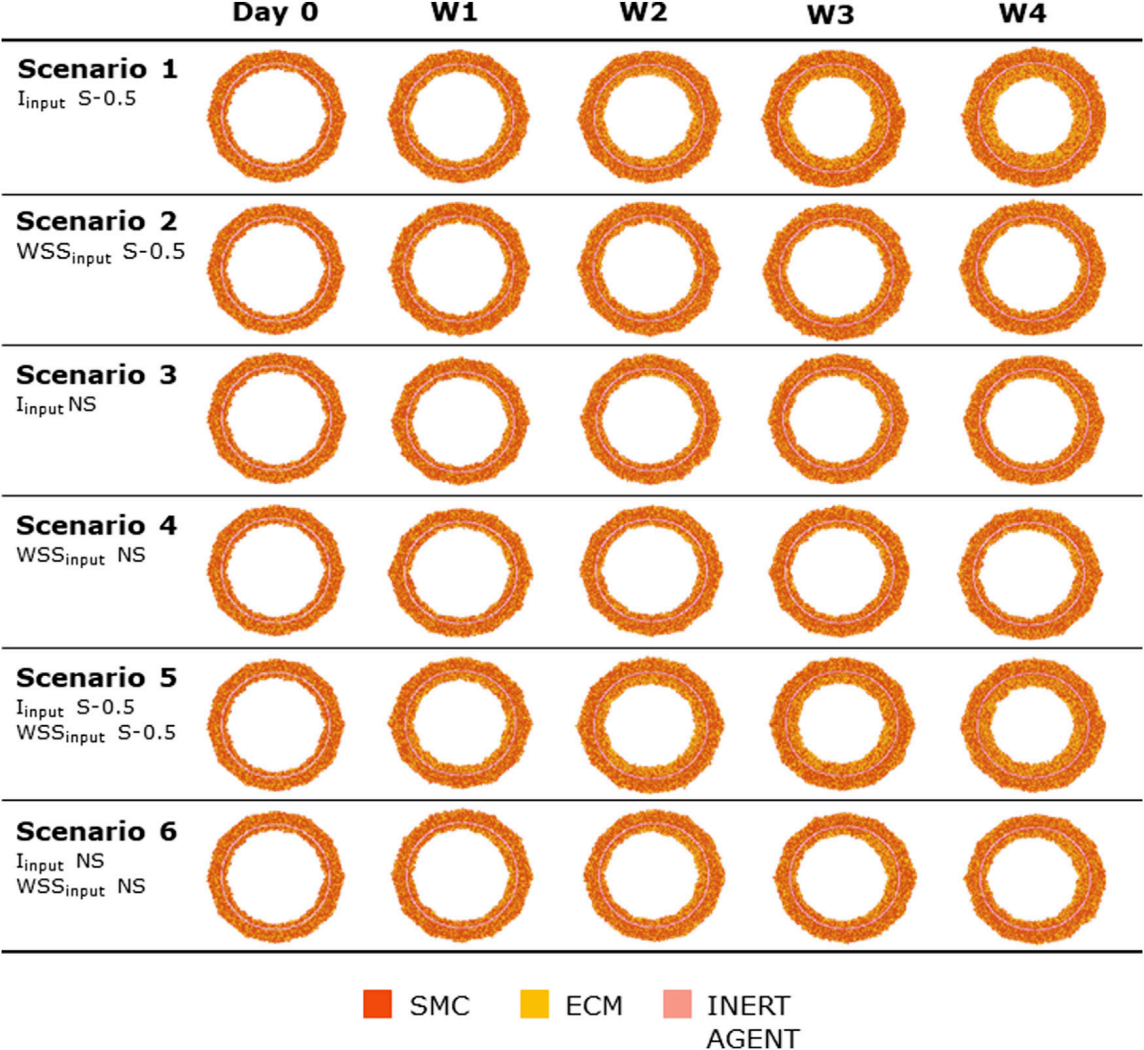
ABM temporal evolution. Simulations under scenarios 1–6 (Table 5) along 4 weeks (Day 0, W1, W2, W3, and W4). For each scenario, the results were retrieved from one out of three ABM simulations, namely, the one presenting the lumen configuration minimizing the root mean square deviation from the average one. W1, week 1; W2, week 2; W3, week 3; W4, week 4; NS, non-symmetric; S, symmetric.

**FIGURE 9:**
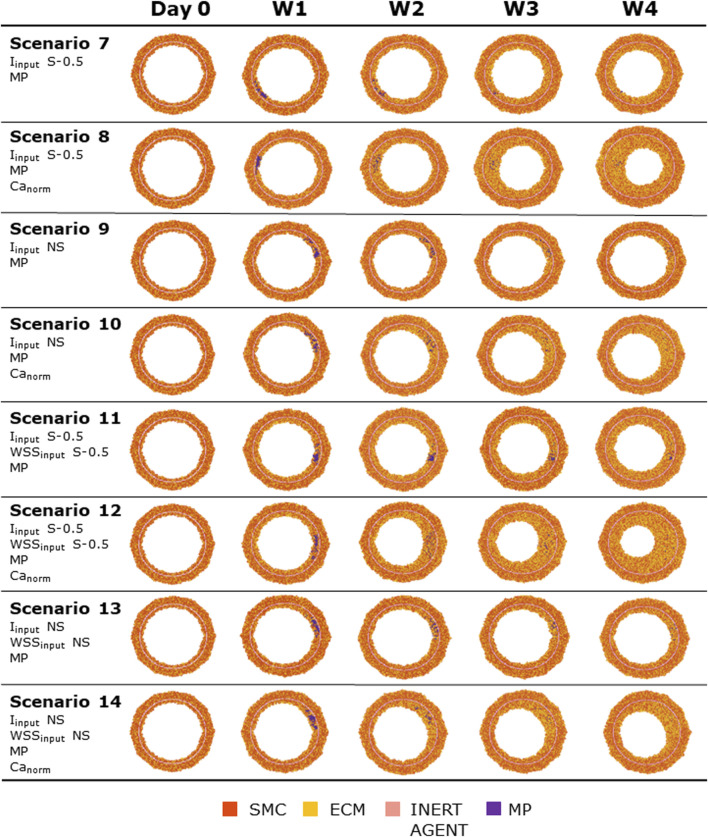
ABM temporal evolution. Simulations under scenarios 7–14 (Table 5) along 4 weeks (Day 0, W1, W2, W3, and W4). For each scenario, the results were retrieved from one out of three ABM simulations, namely, the one presenting the lumen configuration minimizing the root mean square deviation from the average one. W1, week 1; W2, week 2; W3, week 3; W4, week 4; NS, non-symmetric; S, symmetric.

**FIGURE 10:**
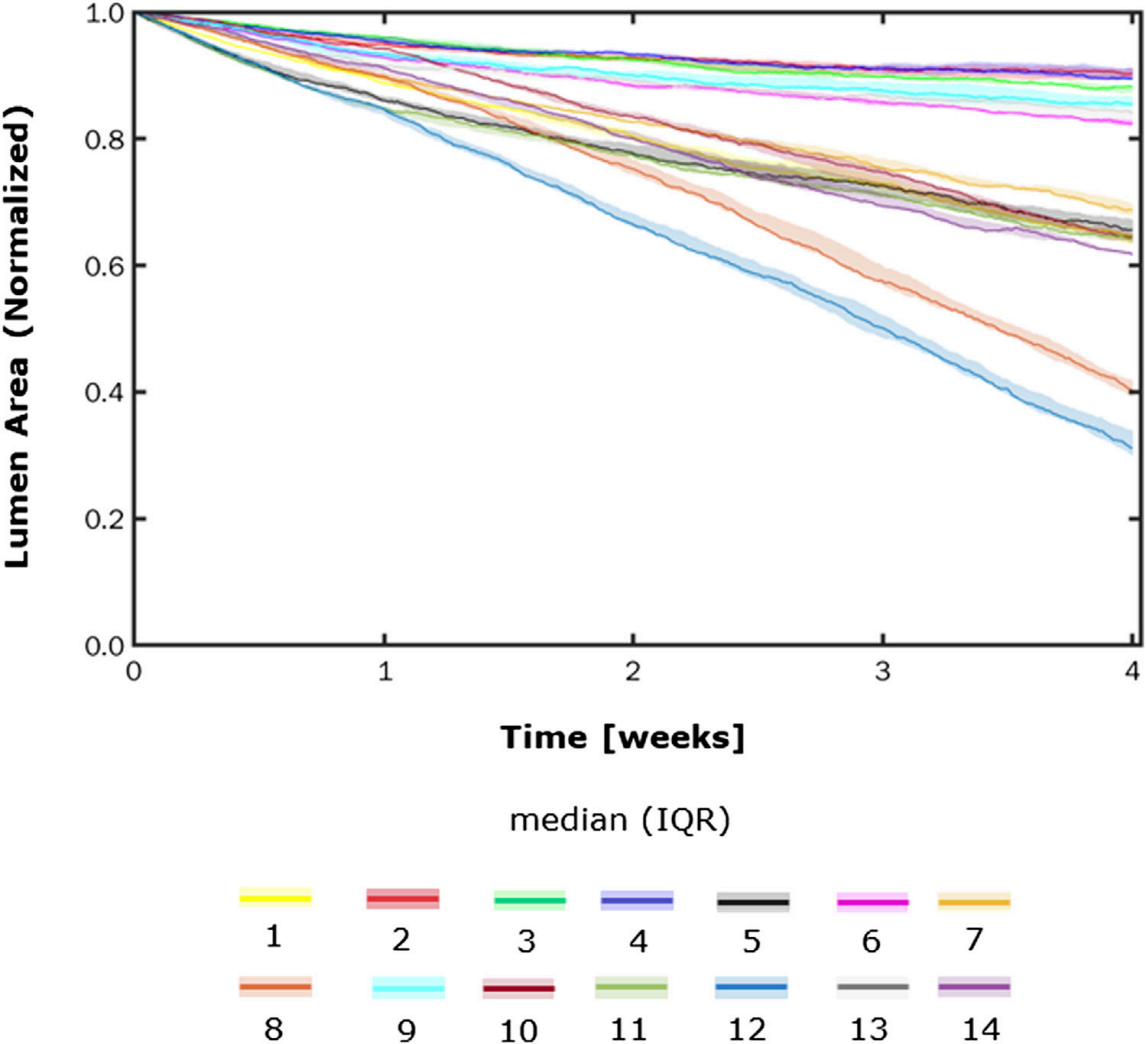
Normalized lumen area vs. time. Simulations under scenarios 1–14 (Table 5) along 4 weeks. The results are provided in terms of the median and interquartile range (IQR) obtained from three repetitions of each scenario.

Figures 8, 9 show the 4-week evolution of the ABM cross-section of the single-input and combined-input scenarios (1–4 and 5–6 in Table 5), and the combined input-event scenarios (7–14 in Table 5), respectively. For each scenario, the representative output cross-section was retrieved among the three repetitions, as anticipated in Section 2.1. Specifically, we computed the mean of the three lumen configuration outcomes and selected the result that exhibited the closest proximity to this average, determined by the mean squared error criterion, as detailed in Corti et al (2020). Figure 10 presents the corresponding normalized lumen area over the time (as median and IQR) of all the 14 scenarios. As presented in Figures 8–10: i) a greater intimal thickening was observed in correspondence of a low WSS or a high $I_{input}$ ; ii) more pronounced occlusions occurred also when events were activated compared to the input-only scenarios; and iii) $I_{input}$ influenced the final intimal thickness more than ${WSS}_{input}$ *,* confirming the findings of the sensitivity analysis.

More in detail, a slight NS intimal growth was obtained under single- and combined NS-input scenarios (scenarios 3, 4, and 6 in Figure 8) and in the presence of single- and combined NS-input with the solo MP infiltration (scenarios 9 and 13 in Figure 9), while a more pronounced NS geometry was observed when both the events were considered (scenarios 10 and 14 in Figure 9), due to the concurrent effects of MP cluster formation and chemoattractant diffusion. This qualitative observation was confirmed by the quantitative calculation of a 4-week NS-index i.e., the percentage ratio between the difference of maximum/minimum lumen radius expressed in polar coordinates and the initial lumen radius of 40 sites, from which we set a threshold of a high NS lumen configuration of 20% (scenario 3: NS-index = 14.78%, scenario 4: NS-index = 12.80%, scenario 6: NS-index = 13.88%, scenario 9: NS-index = 15.70%, scenario 13: NS-index = 15.85%, scenario 10: NS-index = 38.73%, and scenario 14: NS-index = 42.05%, listed in Table 6). As expected, with NS inputs, MP infiltration occurred in correspondence to the peak of $I_{input}$ . A concentric and S intimal thickening was generated under single- and combined S-input scenarios (scenarios 1, 2, and 5 in Figure 8) and in the presence of single- and combined S-input scenarios with the solo MP infiltration (scenarios 7 and 11 in Figure 9), while the scenarios involving both the events (scenarios 8 and 12 in Figure 9) produced a highly NS intimal distribution in correspondence of the chemoattractant release. The NS growth pattern in the S-input scenarios with both events (scenarios 8 and 12 in Figure 9) formed because of the presence of MP clusters, which localized randomly in the cross-section, determining chemoattractant diffusion, in turn promoting cell activation. MP clusters disrupted over time because of both the effect of SMC media-to-intima migration (activated in the presence of the chemoattractant, i.e., scenarios 8, 10, 12, and 14 in Figure 9) and MP apoptosis at later times. This qualitative analysis was verified by the 4-week NS-indexes minor of 10% and major of 20% for the S and the NS geometries, respectively (scenario 1: NS-index = 5.20%, scenario 2: NS-index = 7.15%, scenario 5: NS-index = 5.00%, scenario 7: NS-index = 7.90%, scenario 11: 7.88%, scenario 8: NS-index = 22.95%, and scenario 12: NS-index = 35.85%, listed in Table 6).

**TABLE 6:** NS-index for scenarios 1–14 at 4 weeks.

	Minimum *R*_*L*_	Maximum *R*_*L*_	NS-index (%)
Scenario 1	32.52 sites	34.60 sites	5.20
Scenario 2	37.82 sites	40.68 sites	7.15
Scenario 3	35.14 sites	41.05 sites	14.78
Scenario 4	35.98 sites	41.10 sites	12.80
Scenario 5	31.34 sites	33.34 sites	5.00
Scenario 6	34.25 sites	39.80 sites	13.88
Scenario 7	31.79 sites	34.95 sites	7.90
Scenario 8	19.50 sites	28.68 sites	22.95
Scenario 9	34.39 sites	40.67 sites	15.70
Scenario 10	23.90 sites	39.39 sites	38.73
Scenario 11	31.03 sites	34.18 sites	7.88
Scenario 12	15.83 sites	30.17 sites	35.85
Scenario 13	34.01 sites	40.35 sites	15.85
Scenario 14	22.86 sites	39.68 sites	42.05

*R*_*L*_, lumen radius, computed as the distance between the lumen border and the cross-section center in ABM sites; NS, non-symmetric.

In all the scenarios, the lumen area temporal trend exhibited a linear decrease due to the time-independence of $I_{input}$ and ${WSS}_{input}$ . As shown in Figure 10, three classes of artery response to the scenarios were identified, characterized by i) a slight lumen area decrease (<20%), ii) a moderate lumen area decrease (20%–40%), and iii) a severe lumen area decrease (>50%). The repeatability of the ABM solution was confirmed by the small IQR (Figure 10) obtained from the three repetitions for each scenario and the similarity of the ABM outputs at the 4-week follow-up (Supplementary Figures S4, S5).

### 3.3 CAV manifestations

**FIGURE 11:**
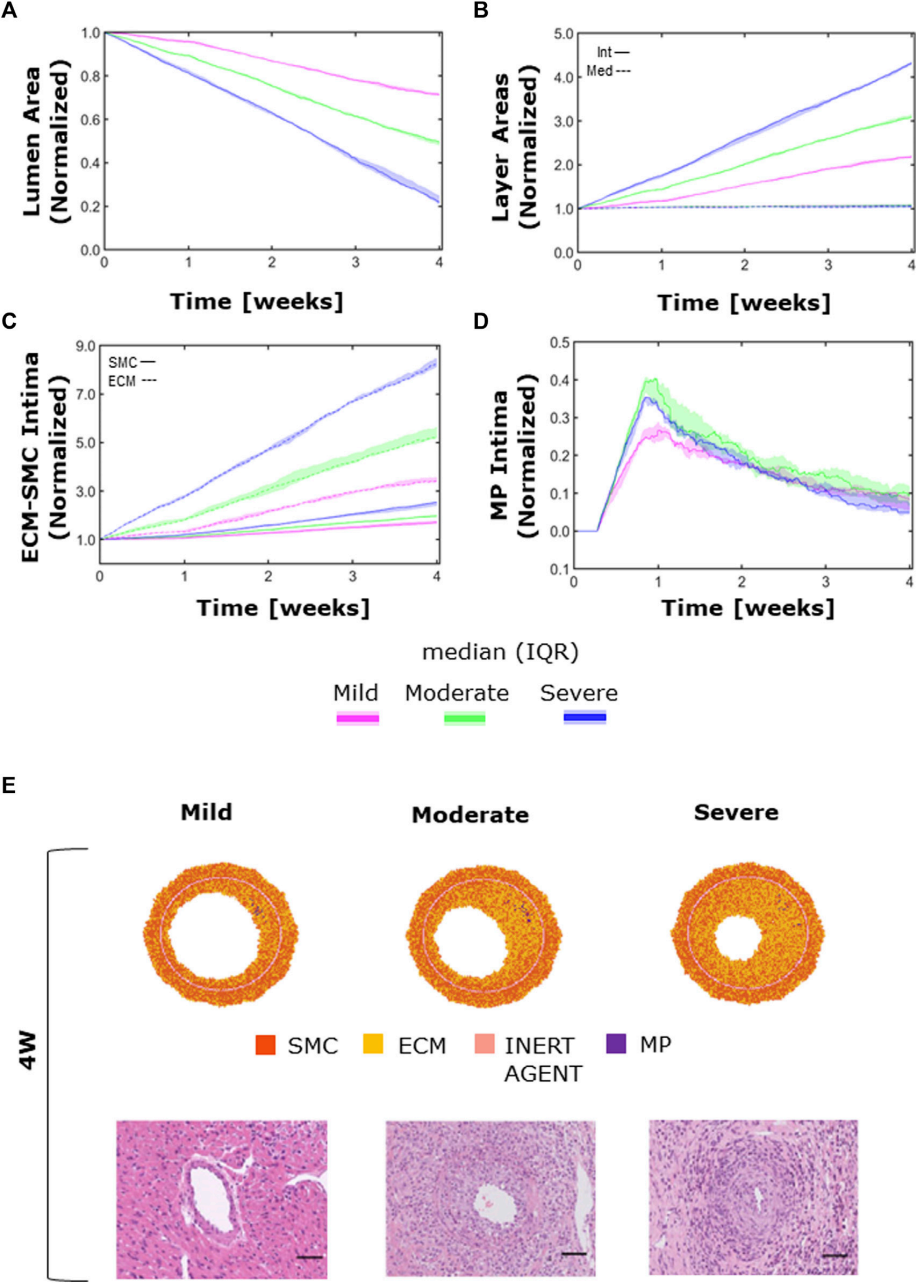
CAV temporal dynamics and qualitative validation. Temporal trends of **(A)** the lumen area; **(B)** intimal (solid line) vs. medial layer (dashed line); **(C)** SMC (solid line) vs. ECM (dashed line) intimal content; **(D)** MP intimal content; **(E)** qualitative validation: comparison between 4-week agent-based model (ABM) outputs and 4-week histological images of cardiac heterotopic transplantation mice model by Zhang et al. (2021) in mild- (left), moderate- (center), and severe (right)-CAV condition. Adapted with permission from Zhang et al. (2021) (https://creativecommons.org/licenses/by/4.0/).

The ABM exhibited heterogeneous CAV degree responses to mild, moderate, and severe NS— $I_{input}$ and NS— ${WSS}_{input}$ . A monotonic decrease of the lumen area was observed in all cases, reaching up to 30%, 50%, and 80% lumen area reduction in the mild-, moderate-, and severe-CAV cases, respectively (Figure 11A). The lumen area reduction reflected the increase of the intima area (Figure 11B), due to the increase in SMC and ECM intima content (Figure 11C), while the media layer preserved the initial area (Figure 11B), due to the absence of perturbed cellular activities in the ABM implementation.

Moreover, the ECM intima content exhibited a higher growth rate than the SMC intima content in all the three CAV cases (Figure 11C). This led to a change in the ECM/SMC relative composition: from an initial hypercellularity (60% SMC and 40% ECM) to a progressive hypocellularity at 4 weeks (56% SMC and 44% ECM in the mild-CAV case, 48% SMC and 52% ECM in the moderate-CAV case, and 42% SMC and 58% ECM in the severe-CAV case).

As regards MP, the temporal trend was characterized by an initial growth within 1 week and a decrease until the end of the 4 weeks, consistently with the ABM implementation explained in Section 2.1.2.1 (Figure 11D). Moreover, the MP content appears to be unrelated to the CAV degree from 1 week because apoptosis is promoted, and therefore MPs are irrelevant for the final areas (i.e., lumen and intima area), which are consequently determined only by the SMC and ECM contents. Figure 11E shows a qualitative comparison between the 4-week ABM representative outputs (selected among three repetitions, as Supplementary Figures S6) and the 4-week histological images of the cardiac heterotopic transplantation mice model without immunosuppressive treatment (Zhang et al., 2021). Both the ABM outputs and the histological images exhibit three degrees of CAV with comparable occlusions. However, the model produced more pronounced NS geometries (especially in the mild and moderate cases) compared to the histological images. Moreover, in the histological images, a greater and less localized content of MPs can be appreciated compared to the ABM cross-section outputs.

## 4 Discussion

The current study presents the first ABM-based approach to study CAV, contributing to a better understanding of this key pathology within chronic HTx rejection. Mouse-based *in vivo* models are considered the gold standard for CAV experimental studies in pre-clinical trials. Motivated by i) the advancements obtained by *in silico* modeling in the cardiovascular field, ii) the promising results of ABMs in deciphering mechanobiological mechanisms driving vascular adaptation, and iii) the suitability of computational models in providing high granularity of investigation time points with very limited resource consumption, this work proposed a 2D ABM of CAV initiation and progression in a mouse LCA model at the cell/tissue scale. The ABM was able to effectively replicate a 4-week CAV initiation and progression, characterized by lumen area decrease due to progressive intimal thickening in regions exposed to high inflammation and low WSS. While hemodynamic-based vascular remodeling processes were already simulated in previous ABMs by our research group (Garbey et al., 2017; Corti et al., 2019; Corti et al., 2020; Corti et al., 2022a; Corti et al., 2022b; Corti et al., 2023b; Corti et al., 2023a), the explicit modeling of inflammatory processes is the key novelty of the present work, being crucial to capture CAV-related phenomena. In particular, the simulated inflammatory cascade encompassed macrophage infiltration in the arterial wall and subsequent mitosis/apoptosis and chemoattractant release, in turn affecting SMC activity. To the best of the authors’ knowledge, except for Bhui and Hayenga (2017), who focused on atherosclerosis, the present study proposes the first ABM of vascular maladaptive phenomena in which the effect of the inflammatory input on immune cell dynamics (e.g., macrophage infiltration) is discerned from other triggers (e.g., hemodynamics) and explicitly simulated.

As emerged through the sensitivity analysis, the inflammatory input constituted the main trigger compared to the WSS-based one. This was consistent with the CAV inflammatory nature and suggested that WSS alone does not support the development of CAV. Moreover, CAV development and progression was governed by the synergic interplay of cellular and molecular events derived from the inflammatory input (MP infiltration and dynamics, chemoattractant diffusion, and SMC media-to-intima migration) rather than by one specific event. The identification of the driving parameters allowed not only gaining insights into the working mechanisms of the ABM but also laying the basis for a future calibration against *ad hoc in vivo* data. Indeed, the primary role played by inflammatory-related events suggests that a future quantitative calibration of the ABM should primarily focus on the inflammatory coefficients found to be statistically significant in driving CAV. As demonstrated in Corti et al. (2022b), this would enable a more efficient calibration, guaranteeing a good accuracy with low computational costs.

The model was also sensitive to different inputs (i.e., S or NS $I_{input}$ and ${WSS}_{input}$ ) and activation/deactivation of events (i.e., MP and chemoattractant diffusion). Three main aspects emerged. First, the influence of inflammation-related factors was dominant with respect to the hemodynamic WSS-based ones, in agreement with the sensitivity analysis results. Second, the ABM developed NS intimal growth patterns not only under NS stimuli, as expected, but also under S ones. In particular, the NS growth was associated to the chemoattractant release, and thus it occurred at the region of MP infiltration. The region of MP infiltration and subsequent chemoattractant release corresponded to the most perturbed region in the case of the NS stimuli, while it was random in the case of the S stimuli. Third, the outcomes of the 14 different scenarios could be grouped into three macro-responses, characterized by three degrees of lumen area occlusion over time (i.e., mild, moderate, and severe). This suggested that activating/deactivating some inputs/events determines similar responses.

The ABM was able to effectively replicate three degrees of CAV under different levels of inflammatory and hemodynamic stimuli. Similar to the histological findings reported by Zhang et al. (2021), the simulated CAV development manifested as a progressive lumen area reduction due to intimal growth determined by the increased SMC and ECM content in the intima. Conversely, in the ABM, MPs did not contribute to intimal area increase: their intimal content over time was characterized by an initial increase, due to infiltration and mitosis (which was favored up to 1 week), followed by a progressive decrease, leading to scarce presence of MPs in the intima at the 4-week follow-up. Although not remarkably contributing to the intimal growth, a greater MP intimal content was present in the histological images, suggesting the need for calibrating the MP dynamics to reflect the histological findings. Moreover, the ABM ECM/SMC relative composition changed during CAV progression, from an initial hypercellularity to a progressive hypocellularity at 4 weeks. However, due to the lack of literature data and the inability to detect ECM/SMC composition in the histological images, at the current stage it was not possible to calibrate the model and assess the reliability of this behavior.

Although minor discrepancies between the model outputs and the histological images were found, the ABM was able to capture major CAV characteristics, thus representing a viable proof-of-concept of CAV in an idealized 2D artery.

The study is not exempt from limitations. First, being a preliminary and methodological investigation, an idealized vessel cross-section geometry was considered. This enabled a robust analysis, allowing to test and refine the model, before applying it to a more realistic vessel geometry. Second, i) SMCs and MPs were the only two cellular phenotypes considered in the ABM, with SMCs being the major arterial wall components and MPs the major players (among the graft-infiltrating cells) in chronic rejection (Vergani et al., 2013; Liu et al., 2016; Lee et al., 2018) and ii) a generic molecule was assumed to represent the action of different released factors (e.g., IFN-γ, IL-2, and TNF-α) on vascular cells. Although simplified, these assumptions allowed qualitatively replicating the pattern of graft-infiltrating cells reported in the literature (McLean et al., 1997; Liu et al., 2016; Ordikhani et al., 2020) and the chemoattractant effect of molecules released by the inflammatory cells. Third, only the WSS was considered a hemodynamic factor, which resulted in not being relevant for CAV development. In future, both the direct and indirect role of hemodynamics in CAV development will be addressed. Regarding the direct role, other hemodynamic descriptors in addition to WSS magnitude (Candreva et al., 2022) could be considered. Regarding the indirect role, the blood transport of immune cells could be investigated. Finally, herein, a qualitative comparison of the model outputs with histological data from the literature was performed due to the lack of experimental available data. Future efforts must be focused on calibrating and validating the model, based on *in vivo* data (vessel geometry, cellular and molecular profiles, etc.).

Although constituting a proof-of-concept, this study demonstrated the potentiality of the developed ABM in gaining further insights in the vasculopathy. Once calibrated and validated, the ABM can be used to support the *in vivo* research, for example, by i) representing a generator of hypotheses of CAV-promoting mechanisms, in turn driving experimental analyses; ii) constituting a digital twin of experimental setups, allowing quantifying processes and variables hardly retrievable *in vivo*; and iii) preliminary testing anti-chronic rejection treatments.

## 5 Conclusion

In this work, an ABM simulating CAV was developed. The proposed ABM effectively replicated a 4-week CAV initiation and progression dynamics in an idealized LCA mouse cross-section. The model was driven by inflammatory and WSS-based stimuli, triggering MP infiltration and subsequent chemoattractant diffusion and perturbed vascular cell activities. The ABM captured relevant CAV features, including: i) decrease in the lumen area and progressive intimal thickening in regions subjected to high inflammation and low WSS, ii) S and NS occlusions mirroring the different experimental outcomes, and iii) the predominant role of inflammation rather than WSS in CAV initiation and progression, confirming the inflammatory nature of the vasculopathy. Our model lays the foundation toward an experimental–computational integrated analysis of CAV, including calibration and validation processes on mouse datasets, to deepen the understanding of the pathology and potentially optimize anti-chronic rejection treatments.

## Data Availability

The original contributions presented in the study are included in the article/Supplementary Material; further inquiries can be directed to the corresponding author.

## Abbreviations

ABM, agent-based model; CAV, cardiac allograft vasculopathy; ECM, extracellular matrix; HTx, heart transplant; IEL, internal elastic lamina; LCA, left coronary artery; MP, macrophage; PRCC, partial rank correlation coefficient; SMC, smooth muscle cell; WSS, wall shear stress.
