# Supplementary material for: An agent-based model of cardiac allograft vasculopathy: toward a better understanding of chronic rejection dynamics
Source: Front Bioeng Biotechnol. 2023 Sep 12;11:1190409. doi: 10.3389/fbioe.2023.1190409 (PMC10523786; doi:10.3389/fbioe.2023.1190409)
Supplement: Supplementary file 1 [file DataSheet1.PDF]

(A)

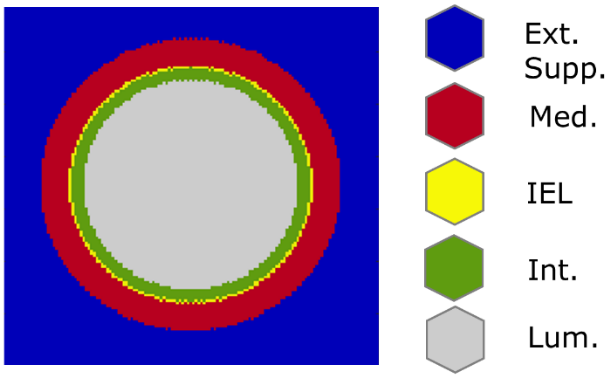

(B)

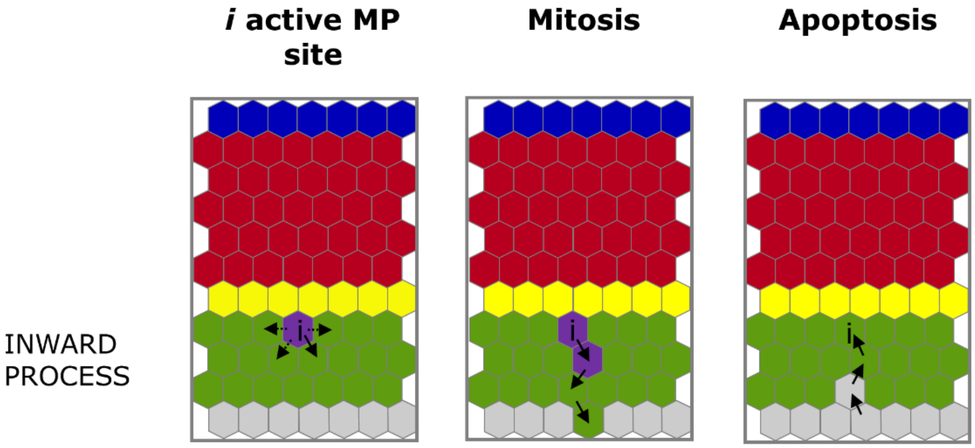

Figure S1. Macrophage (MP) dynamic sequence. (A) Artery cross-section with the compartments, namely lumen, intima, internal elastic lamina (IEL), media, and external support; (B) Left panel: active MP site (i) – Central pannel: site (i) goes in mitosis according to Monte Carlo method and places a daughter MP in a neighbor site or – Right pannel: site (i) goes in apoptosis and removes the MP content in site (i) producing a lumen area variation.

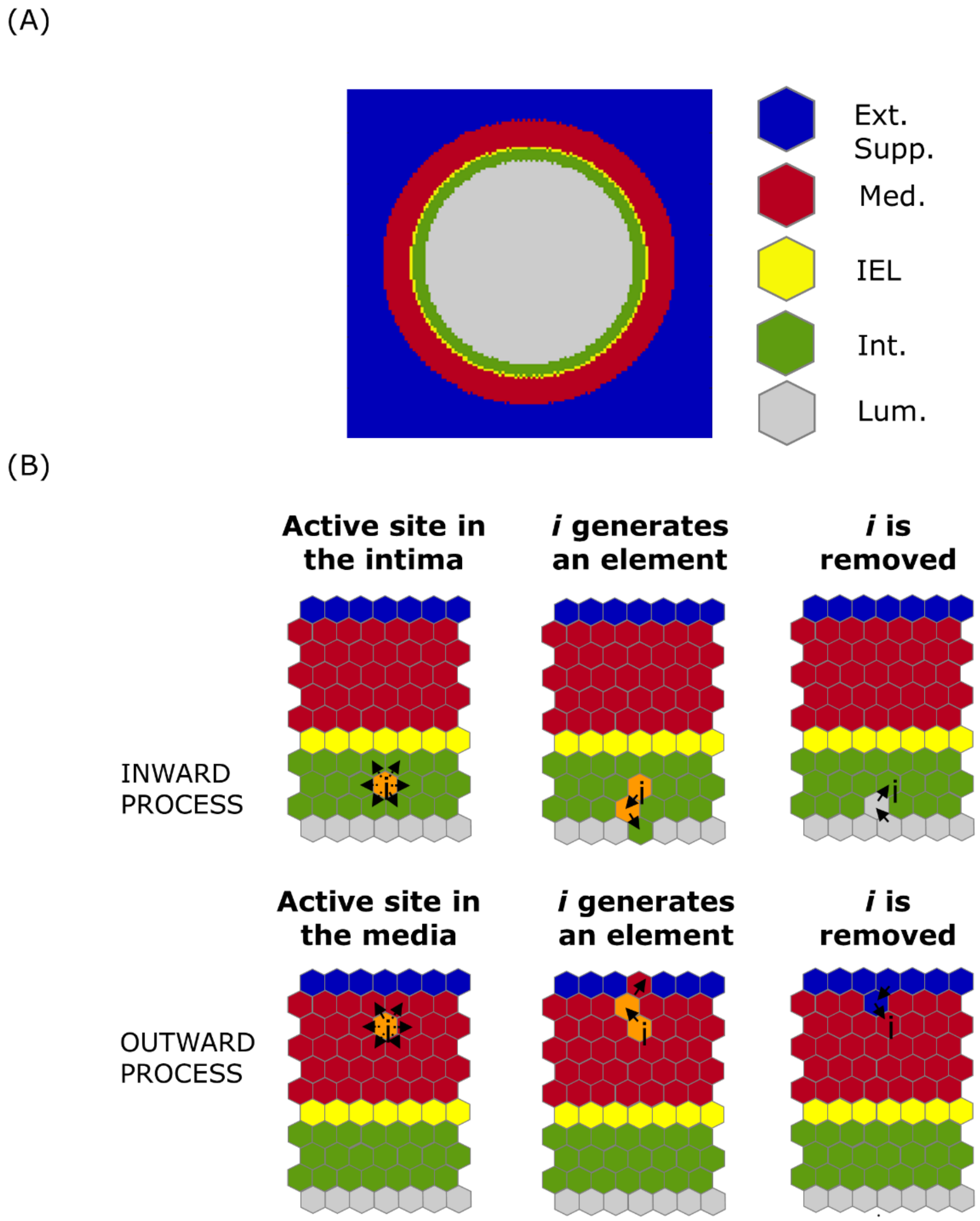

Figure S2. Smooth muscle cell (SMC) and extracellular matrix (ECM) dynamic sequence. **(**A) Artery cross-section with the compartments, namely lumen, intima, internal elastic lamina (IEL), media, and external support; (B) First row shows the inward oriented process. Left panel: if active site (i) in the intima layer goes in – Central panel: mitosis/deposition, a new element is generated and placed in a neighbor site thanks to a movement towards the lumen, – Right panel:

apoptosis/degradation, the hole left by the removing site is filled by an agent movement toward the external support. Second row shows the outward oriented process. Left panel: if active site (i) in the media layer goes in – Central panel: mitosis/deposition, a new element is generated and placed in a neighbor site thanks a movement towards the external support, – Right panel: apoptosis/degradation, the hole left by the removing site is filled by an agent inverse movement toward the lumen.

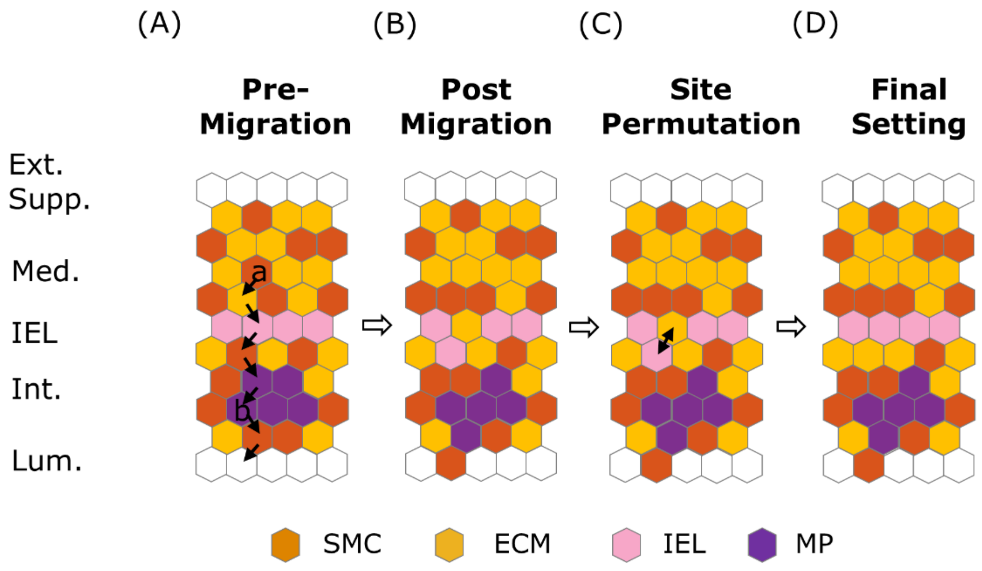

Figure S3. Internal elastic lamina (IEL) regularization process. (A) SMC media-to-intima migration path with starting site (a), ending site (b); (B) Disposition of the inert agent sites showing IEL disruption; (C) permutation between the inert agent site in the intima layer and a SMC site (or ECM site) on the IEL radius; (D) IEL final setting thanks to the regularization algorithm.

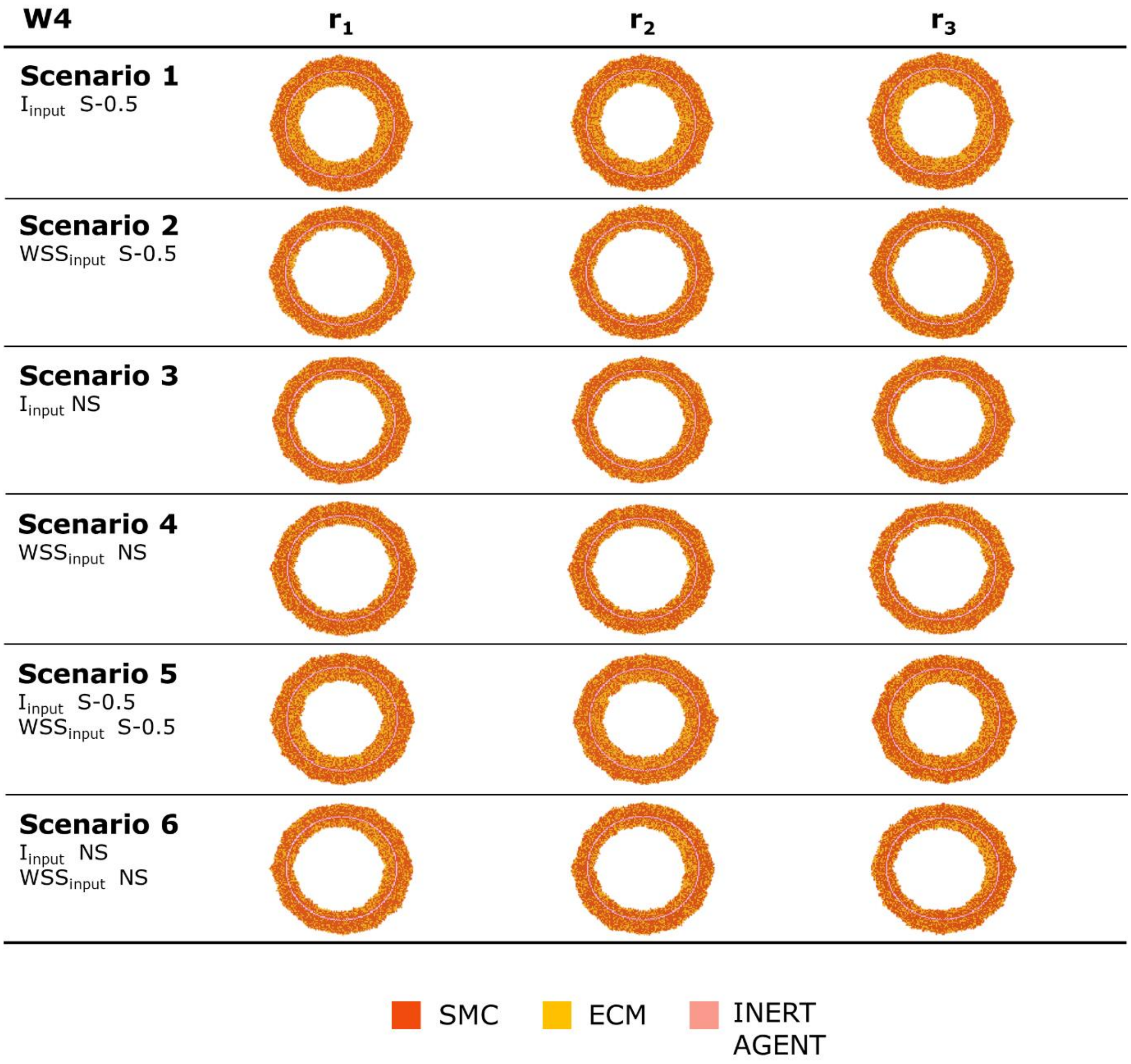

Figure S4. Cross sections of the three simulations for scenarios 1-6 at four weeks (W4). r_1: repetition number 1, r_2: repetition number 2, r_3: repetition number 3.

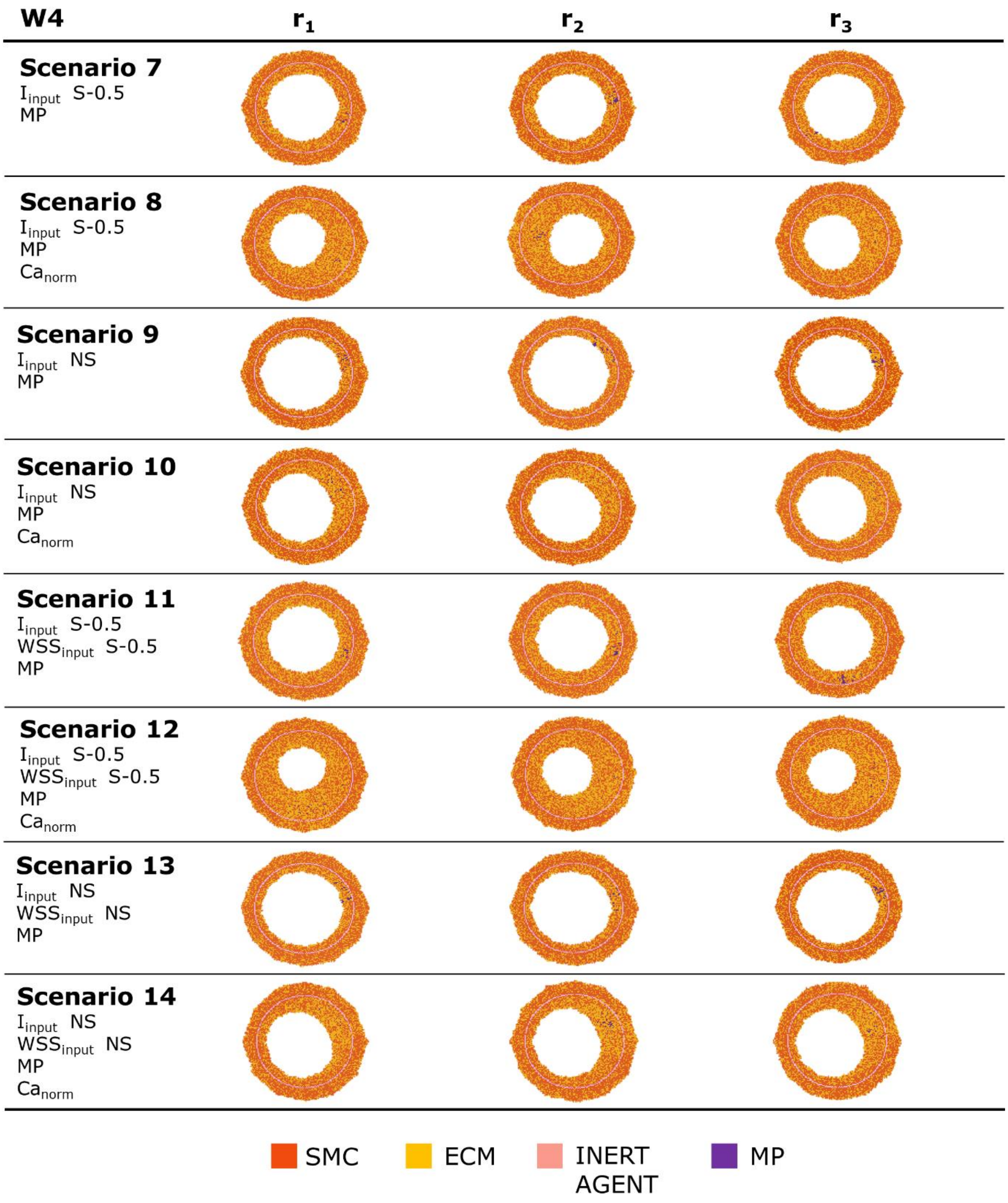

Figure S5. Cross sections of the three simulations for scenarios 7-14 at four weeks (W4). r_1: repetition number 1, r_2: repetition number 2, r_3: repetition number 3.

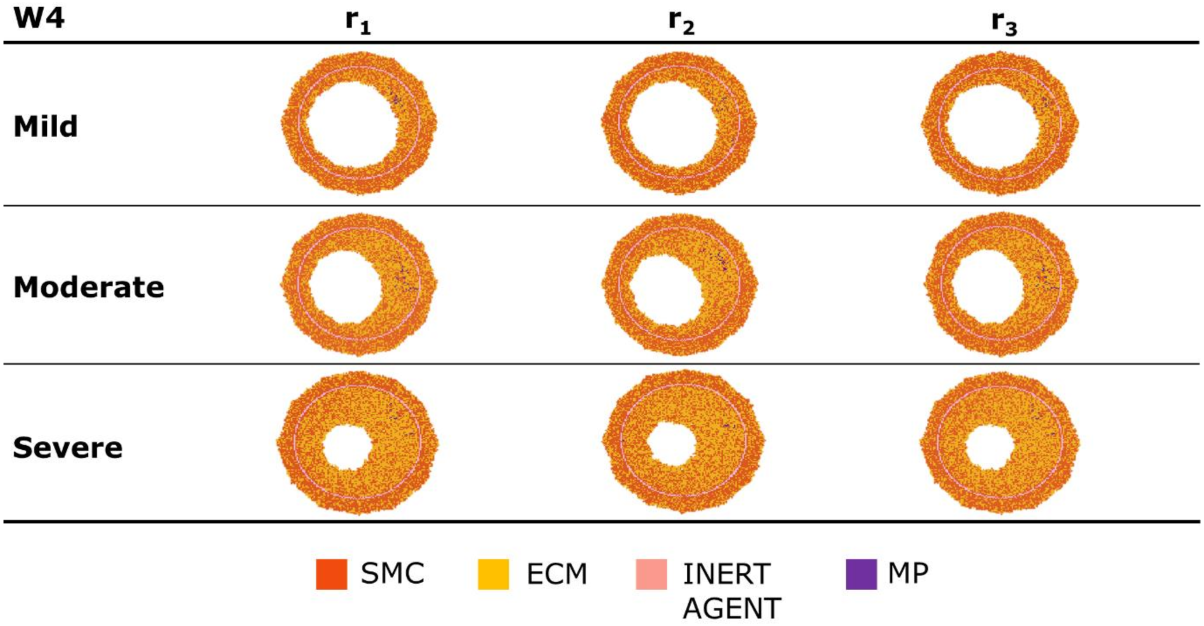

Figure S6. Cross sections of the three simulations for mild, moderate and severe CAV cases at four weeks (W4). r_1: repetition number 1, r_2: repetition number 2, r_3: repetition number 3.
